# Type I Interferon Signaling Augments Autoimmunity in Neuromyelitis Optica Spectrum Disorder

**DOI:** 10.1002/advs.202500942

**Published:** 2025-07-21

**Authors:** Tian‐Xiang Zhang, Xiaoxiao Yang, Xue Gao, Xiaoshan Du, Xuegan Lian, Naiyuan Shao, Ye Liu, Zhenning Huang, Dongmei Jia, Alexander Y. L. Lau, Zhiguo Li, Zaal Kokaia, Fu‐Dong Shi, Chao Zhang

**Affiliations:** ^1^ Department of Neurology Tianjin Medical University General Hospital Tianjin 300052 China; ^2^ Department of Bioinformatics School of Basic Medical Sciences Tianjin Medical University Tianjin 300070 China; ^3^ Department of Neurology The Third Affiliated Hospital of Soochow University Changzhou Jiangsu 213003 China; ^4^ Department of Neurosurgery The Third Affiliated Hospital of Soochow University Changzhou Jiangsu 213003 China; ^5^ Division of Neurology Department of Medicine and Therapeutics, Faculty of Medicine The Chinese University of Hong Kong Shatin Hong Kong 999077 China; ^6^ Laboratory of Stem Cells and Restorative Neurology Lund Stem Cell Center Lund University Lund 22184 Sweden; ^7^ Department of Neurology China National Clinical Research Center for Neurological Diseases Beijing Tiantan Hospital Capital Medical University Beijing 100070 China; ^8^ State Key Laboratory of Experimental Hematology National Clinical Research Center for Blood Diseases Haihe Laboratory of Cell Ecosystem Tianjin 300052 China

**Keywords:** innate immune response, neuromyelitis optica spectrum disorder, T cells, type I interferon

## Abstract

Neuromyelitis optica spectrum disorder (NMOSD) is an autoimmune disease characterized by anti‐aquaporin 4 (AQP4) antibody‐mediated astrocyte damage and subsequent demyelination. Prior attempts to treat NMOSD with interferon‐beta (IFN‐β), a disease‐modifying therapy for multiple sclerosis, resulted in worsening of disease activity, with an unknown mechanism. Here, robust activation of the cGAS‐STING‐IFN‐I signaling pathway is identified in myeloid cells in both the periphery and central nervous system. The abnormal IFN‐I response gives rise to an increase in the number of AQP4 antigen‐specific autoreactive T cells. *Sting* deficiency can significantly blunt the activation of AQP4‐specific T cells, as well as the IFN‐I activity in microglia, and attenuate astrocyte damage. Consequently, the clinical manifestation of NMOSD is ameliorated in a passive transfer mouse model of NMOSD. Further, treatment with STING inhibitor H151 alleviates the severity of NMOSD mouse models. These findings uncover the cGAS‐STING‐IFN‐I pathway in promoting autoreactive T cells and establish a foundation for inhibiting this pathway as a new therapeutic revenue for NMOSD.

## Graphical abstract

The cGAS‐STING innate immune pathway is excessively activated both in peripheral monocytes and CNS microglia in patients with NMOSD, leading to an overproduction of IFN‐I. IFN‐I subsequently acts on AQP4‐specific autoreactive T cells. Blocking this pathway may offer novel therapeutic options for individuals with NMOSD.

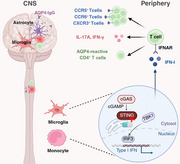

## 1 Introduction

Neuromyelitis optica spectrum disorder (NMOSD) is an autoimmune disease affecting the central nervous system (CNS), characterized by the presence of autoantibodies against aquaporin‐4 (AQP4‐IgG) in majority of patients.^[^ ^1^ ^]^ AQP4‐IgG, generated by autoreactive B cells, directly damages astrocytes through binding to astrocytic endfeet.^[^ ^2^ ^]^ Several studies have highlighted the crucial role of the innate immune response in NMOSD pathogenesis. Specifically, as CNS resident macrophages, microglia likely serve as primary responders to astrocytes upon activation by AQP4‐IgG. The presence of activated microglia around NMOSD lesion sites can potentially initiate innate immune responses, resulting in infiltration of inflammatory cells, complement deposition, and neuronal and oligodendrocyte damage within the CNS.^[^ ^3^, ^4^ ^]^

Interferon‐beta (IFN‐β) subtypes have been used as immunomodulatory agents for relapsing‐remitting multiple sclerosis (MS).^[^ ^5^ ^]^ Paradoxically, IFN‐β therapy exacerbates NMOSD activity,^[^ ^6^, ^7^ ^]^though the underlying mechanism remains elusive. Dysregulated type I interferon (IFN‐I) activity in the blood of NMOSD patients has been associated with an increase in interleukin‐6 (IL‐6) levels, which promote the differentiation of pathogenic Th17 cells.^[^ ^8^ ^]^ Further, aberrations in IFN‐I responses have been observed in both the cerebrospinal fluid (CSF) and peripheral blood B cells of NMOSD patients.^[^ ^9^ ^]^ The presence of IFN‐I also facilitates the differentiation of B cells into antibody‐secreting cells, thereby augmenting AQP4‐IgG production.^[^ ^9^ ^]^ However, the molecular mechanisms underlying abnormal IFN‐I production in NMOSD patients and their role in exacerbating the disease are not fully understood. This study aims to examine the contributions of IFN‐I to NMOSD as well as its molecular underpinnings, and clinical implications.

## 2 Results

### 2.1 Microglia and CNS‐Associated Macrophages Exhibited Enhanced cGAS‐STING‐IFN‐I Activity

**Figure 1:**
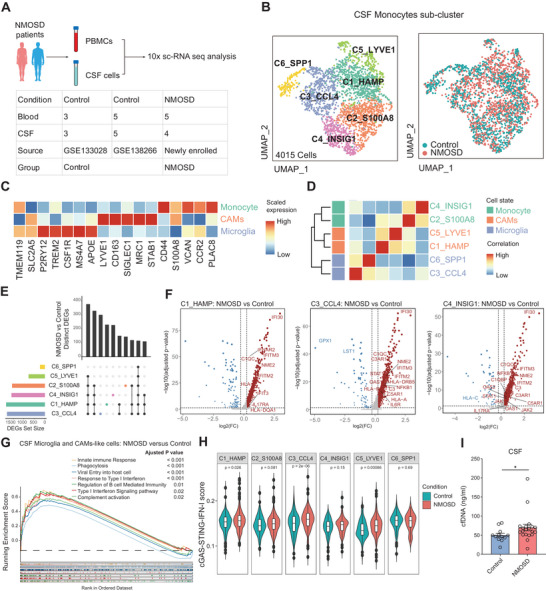
Enhanced cGAS‐STING‐IFN‐I signaling in CSF microglia‐like cells of NMOSD patients. A) Workflow illustrating the study design and sample selection for scRNA‐seq analysis of peripheral blood and CSF. B) Uniform manifold approximation and projection (UMAP) plot illustrating CSF monocytes subclusters based on scRNA‐seq data. C) The expression levels of representative marker genes used to classify microglia‐like cells, CNS‐associated macrophages‐like cells, and monocytes states. D) Correlations between CSF monocytes subclusters in CSF and three cell states: microglia, CNS‐associated macrophages, and monocytes. E) UpSet plot illustrating size and overlap of DEGs in CSF monocytes subclusters between NMOSD patients and controls. F) The top DEGs of the CSF monocyte and microglia subclusters. G) GSEA plot illustrating selected significantly enriched KEGG pathways in the CSF microglia‐like cells and CNS‐associated macrophages‐like cells between control individuals and NMOSD patients. H) cGAS‐STING‐IFN‐I gene‐set score in the CSF monocyte and microglia subclusters, comparing NMOSD patients with controls using the Wilcoxon rank‐sum test. I) The levels of cfDNA in CSF were detected from both control and patients with NMOSD. (control = 12, NMOSD = 20). Data were analyzed using Mann Whitney test; Mean ± s.e.m; * *p* < 0.05. CAMs, CNS‐associated macrophages.

Paired cerebrospinal fluid (CSF) and peripheral blood mononuclear cells (PBMCs) samples were collected from five NMOSD patients with treatment‐naive AQP4‐IgG positive NMOSD for single‐cell RNA sequencing (scRNA‐seq) analysis. In addition, we included a total of 8 paired CSF and PMBCs samples from 3 healthy controls (HC) and 5 pairs of non‐neuroinflammatory samples from two independent scRNA‐seq studies as the control group for subsequent analysis^[^ ^10^, ^11^ ^]^ (**Figure** **1**A; and Table S1, Supporting Information). After implementing a rigorous quality control process and addressing batch effects, we were able to effectively isolate 134, 603 immune cells. We identified main cell types including CD4^+^ and CD8^+^ T cells, T regulatory cells (Tregs), natural killer (NK) cells, B cells, plasma cells, conventional dendritic cells (cDCs), plasmacytoid dendritic cells (pDCs), as well as monocytes through our analysis (Figure S1A–D, Supporting Information). Differential genes expression (DEGs) analysis over different cell types revealed that monocytes and T cells exhibited the most significant gene alterations in both blood and CSF (Figure S1E,G, Supporting Information), with an overlap in inflammation‐related genes (Figure S1F,H, Supporting Information).

Several studies have identified cells resembling microglia and CNS‐associated macrophages‐like cells in the CSF.^[^ ^12^, ^13^, ^14^ ^]^ To further investigate the cellular alterations associated with neuroinflammation in CSF myeloid cells of NMOSD patients, we conducted re‐clustering analysis of monocytes in the CSF (Figure 1B). This analysis revealed three distinct cell subtypes based on their unique molecular markers: two clusters of CNS‐associated macrophages (C1_HAMP and C5_LYVE1, marked by *LYVE1*, *CD163*, and *MRC1*), two clusters of monocytes (C2_S100A8 and C4_INSIG1, identified by *S100A8*, *CCR2* and *VCAN*), and two clusters of microglia (C3_CCL4 and C6_SPP1, characterized by *P2RY12*, *CSF1R*, and *TREM2*), (Figure 1C,D; Figure S2A, Supporting Information). KEGG (Kyoto Encyclopedia of Genes and Genomes) enrichment analysis assessed the functional states of the identified clusters of microglia‐like and CNS‐associated macrophages‐like cells (Figure S2B,C, Supporting Information). C1_HAMP displayed characteristics associated with autoimmune diseases and antiviral immunity terms, such as “Epstein‐Barr virus infection” and “Cytokine‐cytokine receptor interaction”. Other sub‐clusters, like C3_CCL4 and C4_INSIG1, associated with inflammatory responses, displayed by profiles characterized by the expression profile of chemokine and cytokine genes, such as *CCL4*, and *IL1B* (Figure S2B, Supporting Information), and terms like “Th17 cell differentiation” and “antigen processing and presentation” (APC) in the KEGG analysis (Figure S2C, Supporting Information), suggesting their potential roles in immune response regulation. C5_LYVE1 demonstrated typical macrophage phagocytic function and showed elevation in the proportion within CSF of NMOSD patients (Figure S2C,D, Supporting Information).

During the DEGs analysis of each sub‐cluster within the CSF, a significant number of overlapping DEGs were identified among these clusters (Figure 1E). Among them, these genes predominantly included those involved in IFN‐I response, such as *IFNAR1, IFNAR2*, *IFITM2, IFITM3*, as well as *IL17RA*, and complement activation genes *C1QB, C1QC* (Figure 1F). Subsequently, we performed Gene Set Enrichment Analysis (GSEA) for microglia and CNS‐associated macrophages‐like cells in the CSF of NMOSD patients and controls. The results revealed dysregulated activation of IFN‐I‐related pathways, including the “innate immune response”, “type I interferon signaling pathway”, and “viral entry into host cells” in the microglia and macrophages of NMOSD patients (Figure 1G). Given the critical role of the cGAS (cyclic GMP‐AMP synthase)‐STING (stimulator of interferon genes) pathway in inducing IFN‐I production through the recognition of pathogenic DNA nucleic acids, and as a key mechanism for host defense against pathogens,^[^ ^15^, ^16^ ^]^ we conducted a comprehensive analysis of the cGAS‐STING‐IFN‐I score in CSF microglia and macrophages. Our analysis indicated that NMOSD patients exhibited significantly elevated cGAS‐STING‐IFN‐I scores in these CSF clusters, including C1_HAMP, C3_CCL4, and C5_LYVE1, when compared to the control group (Figure 1H). The levels of cell free double stranded DNA (cfDNA) were directly detected in paired CSF and plasma samples from patients with NMOSD and controls. The results indicated that the levels of cfDNA in CSF were significantly correlated with those in plasma (Figure S2E, Supporting Information), and the levels of cfDNA in CSF and plasma of patients with NMOSD were notably higher than those of the control group (Figure 1I; Figure S2F, Supporting Information). Moreover, consistent with DEG analysis results, the pathways of “complement activation” and “B cell‐mediated immune regulation” were also upregulated in NMOSD patients versus control (Figure 1G). Collectively, these findings highlight the significant role of cGAS‐STING mediated IFN‐I production in the CNS compartment and emphasize the involvement of B cells and Th17 cells in the pathogenesis of NMOSD.

### 2.2 cGAS‐STING‐IFN‐I Activation in NMOSD Peripheral Blood Monocytes

**Figure 2:**
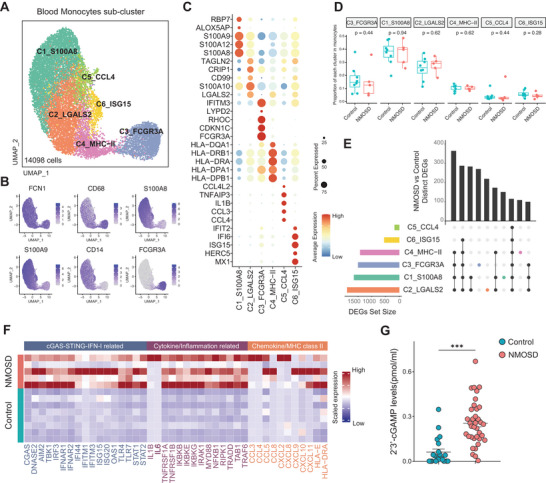
Blood monocytes displayed IFN‐I over‐activity similar to that observed within the CSF of NMOSD patients. A) UMAP plot depicting the subclusters of blood monocytes derived from scRNA‐seq data. B) UMAP plot illustrating the expression patterns of canonical markers in blood monocytes. C) Dot plot illustrating the differential expression of highly expressed genes within distinct subclusters. D) Box plot depicting disparities in the proportions of blood monocyte subclusters between patients with NMOSD and control subjects. E) UpSet plot visualizing the comparative size and overlap of DEGs within blood monocyte subclusters between NMOSD patients and controls. F) Representative IFN‐I and pro‐inflammatory related genes between NMOSD patients and control individuals. G) The plasma levels of 2′3′‐cGAMP obtained from both control and patients with NMOSD were quantified by ELISA. (control = 21, NMOSD = 36). Data were analyzed using Mann Whitney test; Mean ± s.e.m; ***p < 0.001.

Having identified cGAS‐STING‐IFN‐I activation in CSF myeloid cells, we next asked whether similar dysregulation would occur in peripheral blood monocytes. We conducted a re‐clustering analysis of peripheral blood monocytes and successfully identified six distinct subclusters (**Figure** **2**A). C1_S100A8, C2_LGALS2, C5_CCL4, and C6_ISG15 were more likely to be classified as classical CD14^+^ monocytes, while C4_MHC‐II were identified as CD14^+^CD16^+^ intermediate monocytes, and C3_ FCGR3A were categorized as CD16^+^ non‐classical monocytes (Figure 2B). Among them, there were no significant differences in the proportions of these clusters in peripheral blood, and C3_FCGR3A and C5_CCL4 clusters demonstrated heightened pro‐inflammatory functions (Figure 2C,D; Figure S3B, Supporting Information). Consistent with the observations in the CSF, DEGs and GSEA analysis further revealed that monocytes subclusters derived from the blood of NMOSD patients also exhibited a robust cGAS‐STING‐IFN‐I response (Figure 2E,F; Figure S3C,D, Supporting Information). Additionally, we analyzed cGAS‐STING‐IFN‐I scores for identified main immune cell subtypes and found that myeloid cells, particularly, peripheral blood monocytes and CSF microglia‐like cells in NMOSD patients exhibited higher cGAS‐STING‐IFN‐I scores (Figure S4, Supporting Information). We also measured plasma levels of 2′3'‐cGAMP (2′3'‐cyclic GMP‐AMP), a crucial component in the cGAS‐STING pathway that has a strong binding affinity to STING and potent induction of IFN‐I.^[^ ^16^ ^]^ Our findings revealed a significant increase in the levels of 2′3′‐cGAMP within plasma samples in NMOSD patients compared to controls (3.92‐fold increase) (Figure 2G). These findings underscore the role of monocytes and microglia as primary sources of IFN‐I in NMOSD patients and implicate cGAS‐STING‐IFN‐I activation in the disease pathogenesis.

### 2.3 IFN‐I Promotes the Inflammatory Response of T Cells in NMOSD

**Figure 3:**
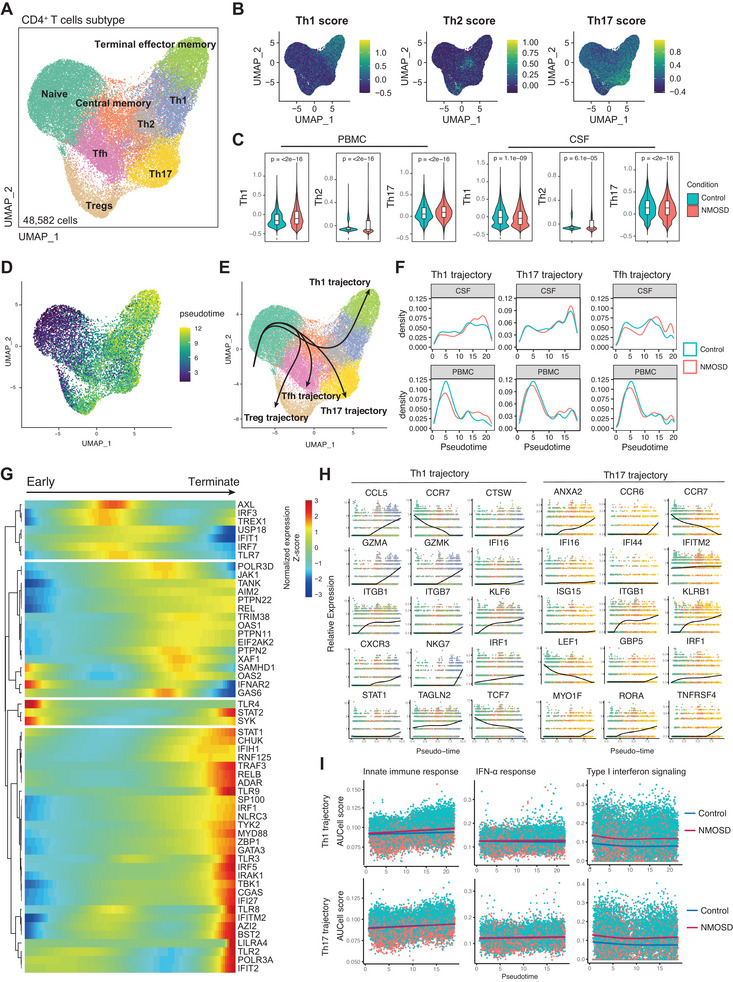
IFN‐I facilitated the development of CD4^+^ T cells in NMOSD. A) UMAP plot depicting the subclusters of CD4^+^ T cells derived from all samples of scRNA‐seq data. B) The gene‐set scores of different T helper subsets (Th1, Th2, and Th17 gene signatures) in CD4^+^ T cells. C) The gene scores of different helper T cell subsets (characterized by Th1, Th2, and Th17 gene signatures) were compared between the control group and patients with NMOSD. D,E) UMAP plot showing pseudotime trajectory of CD4^+^ T cells based on Slingshot demonstrated three distinct trajectories (Th1, Th17, and Tfh). F) The density plot of CD4^+^ T cell differentiation trajectories showing the changes in the intensity of different T cell subtypes during the differentiation process between the NMOSD group and the control group. G) Heat map illustrating the normalized log count of gene expression associated with IFN‐I response in CD4^+^ T cells during the trajectory of differentiation and maturation. H) The expression levels of representative genes driving CD4^+^ T cells development along Th1 and Th17 trajectories. I) The differences in IFN‐I‐related pathway scores between the NMOSD group and the control group during the differentiation process of CD4^+^ T cells.

Our prior findings suggest the potential involvement of microglia and monocytes in the differentiation and maturation process of T cells in NMOSD patients. Subsequently, we carried out a further sub‐cluster analysis of CD4^+^ T cell subsets and defined naive T cells, central memory (CM) T cells, Tregs (C7_FOXP3), follicular helper T cells (Tfh), Th1 cells, Th2 cells, Th17 cells, and terminal effector memory T cells (TEMRA) (**Figure** **3**A; Figure S5A–C, Supporting Information). Subsequently, we verified the scoring of the defined T cell subtypes, thus confirming the classification of Th1, Th2, and Th17 subtypes (Figure 3B). Notably, our results showed that the CSF and peripheral blood of NMOSD patients had higher scores for CD4^+^ T cell subtypes (Figure 3C), along with more robust cellular activation and cytotoxic scores (Figure S5D,E, Supporting Information). These findings indicated a stronger inflammatory response in CD4^+^ T cells of NMOSD patients. We next explored the effect of IFN‐I on the differentiation of CD4^+^ T cells by pseudotime analysis. Initially, CD4^+^ T cells manifested several distinct major differentiation trajectories: from the naive state into the Th1 cell type and subsequently evolved into terminal effector memory T cells; from the naive state into the Th17 cell subtype; from the naive state to the follicular helper T cell state, and from the naive state to the Treg cell state (Figure 3D,E). Among these, CD4^+^ T cells derived from patients with NMOSD demonstrated a higher intensity of differentiation in the middle and late stages of the differentiation process (Figure 3F). Subsequently, we found that the expression of IFN‐I response‐related genes showed a gradually increasing trend during the differentiation of CD4^+^ T cells from the naive state to the terminally effector state (Figure 3G). Specifically, in the Th1 differentiation trajectory, IFN‐I response‐related genes such as *IRF1*, *STAT1*, and *IFI16* showed a significant up‐regulation in the expression from the naive state to the terminal state (Figure 3H). In the Th17 differentiation trajectory, the expression of IFN‐I response‐related genes such as *IFI16*, *IFITM2*, *ISG15*, and *GBP5* gradually increased (Figure 3H). In addition, during the differentiation process, the IFN‐I response‐related pathway score of NMOSD patients was significantly higher than that of the control group (Figure 3I). These findings collectively demonstrate an enhanced inflammatory response in CD4^+^ T cells from NMOSD patients, where elevated IFN‐I activity further amplified the inflammatory responses of CD4^+^ T cells, potentially contributing to disease pathogenesis.

### 2.4 Activation of cGAS‐STING‐IFN‐I Pathway in NMOSD Mouse Models

**Figure 4:**
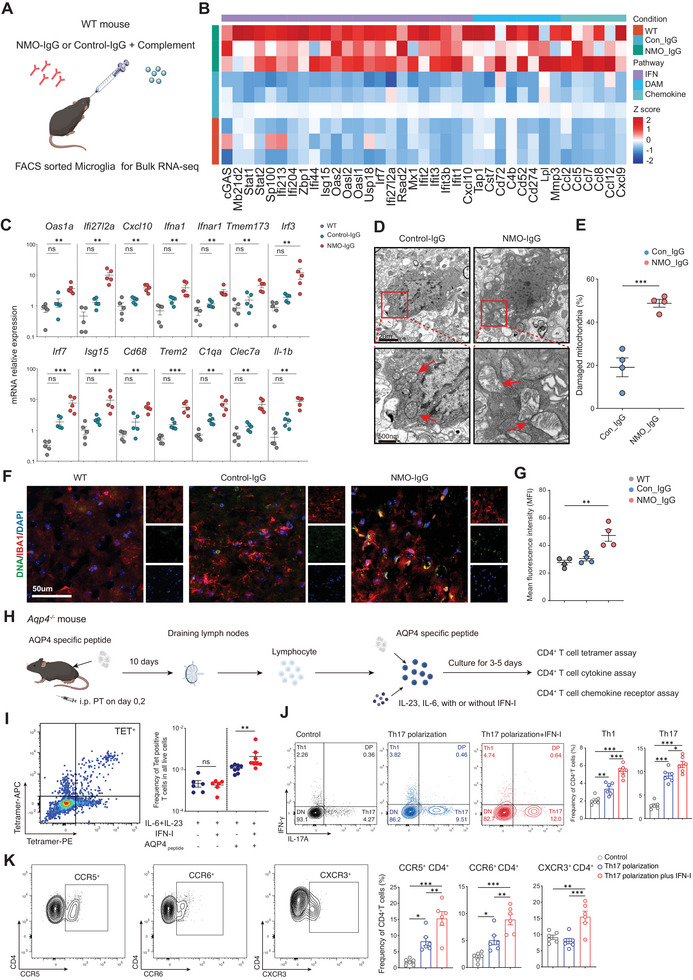
Enhanced cGAS‐STING‐IFN‐I activity in NMOSD Mouse Models. A) Schematic representation of NMO‐IgG injection model. B) Heatmap illustrating the expression levels of cGAS‐STING‐IFN‐I, disease‐associated microglia (DAM), and chemokine genes derived from bulk RNA‐seq of microglia sorted from WT mice, Control‐IgG treated mice, and NMO‐IgG treated mice (n=3 per group). C) Quantitative PCR analysis revealed altered expression for cGAS‐STING‐IFN‐I and microglia activation‐related genes in MACS‐sorted CD11b^+^ microglia from WT mice, Control‐IgG‐treated mice, and NMO‐IgG‐treated mice during the peak of disease (*n* = 5 per group). D,E) Representative transmission electron microscopy images and quantitative analysis of damaged mitochondrial alterations in microglia of control‐IgG‐treated mice and NMO‐IgG‐treated mice (*n* = 4 per group). F,G) Representative immunofluorescence staining images and quantitative analysis of the changes in DNA mean fluorescence intensity within IBA1^+^ microglia in WT mice, control‐IgG‐treated mice, and NMO‐IgG‐treated mice (*n* = 4 per group). H) Flow chart depicting the acquisition and in vitro culture procedure of AQP4‐specific T cells. I) Representative flow cytometry and statistical analysis demonstrated that IFN‐I promoted an increase in the proportion of I‐A^b^‐AQP4(205‐215) tetramer‐positive T cells within CD4^+^ T cells (*n* = 6–8). J) Representative flow cytometry and statistical analysis revealed that under Th17 polarization conditions, IFN‐I promoted the increase in the proportion of IFN‐γ and IL‐17A positive T cells in CD4^+^ T cells (*n* = 6). K) Representative flow cytometry and statistical analysis demonstrated that IFN‐I promoted an increase in the proportion of chemokine receptors CCR5, CCR6, and CXCR3 positive T cells in CD4^+^ T cells (*n* = 6). Data presented as Mean ± s.e.m; *p < 0.05, **p < 0.01, ***p < 0.001, P values were calculated by the one‐way ANOVA test, Kruskal‐Wallis test, or Mann‐Whitney test.

To further confirm the aberrant activation of the cGAS‐STING‐IFN‐I pathway in microglia‐like cells identified in the CSF of NMOSD patients, we first utilized the NMO‐IgG intracerebral injection mouse model (NMO‐IgG model)^[^ ^17^ ^]^ which is capable of mimicking the response of microglia subsequent to the direct attack of NMO‐IgG on astrocytes in the CNS during the pathogenesis of NMOSD. We performed bulk RNA‐sequencing (RNA‐seq) analysis on fluorescence‐activated cell sorted CD45^int^ CD11b^+^ microglia from wild‐type (WT) mice, control‐IgG injected mice, and NMO‐IgG injected mice (**Figure** **4**A). Transcriptional analysis revealed that mice in the NMO‐IgG injected group showed an upregulation of cGAS‐STING‐IFN‐I pathway related genes expression in microglia, including *Cgas*, *Isg15*, and Z*bp1*. This was accompanied by the activation of disease‐associated microglia (DAM) genes^[^ ^18^ ^]^ and an increase in chemokine‐related genes expression (Figure 4B). Meanwhile, qPCR experiments conducted on CD11b^+^ microglia isolated by magnetic cell sorting (MACS) from brain tissue revealed that NMO‐IgG injection resulted in increased expression of genes associated with the cGAS‐STING‐IFN‐I pathway, as compared to WT and control‐IgG injected mice. This included increased expression of *Tmem173* gene (encoding STING), *Irf3*, *Ifna1*, *Ifnar1*, *Cxcl10*, and *Isg15* at the peak of the disease. Activation of microglia activation genes, including *Cd68*, *Il‐1b*, *Clec7a*, and *C1qa* were also upregulated (Figure 4C). As mitochondrial DNA (mtDNA) leakage is also a core activator of the cGAS‐STING signaling pathway,^[^ ^19^ ^]^ we next investigated the changes in mitochondrial homeostasis in NMOSD mouse models using transmission electron microscopy (TEM). We found that the mitochondria in the microglia of mice treated with NMO‐IgG exhibited abnormal morphology, identified by the loss of cristae and/or the disruption of the mitochondrial outer and/or inner membrane (Figure 4D,E). Immunofluorescence analysis also revealed an increase in DNA accumulation within IBA1^+^ microglia in the NMO‐IgG group (Figure 4F,G). The key protein IRF3 in the cGAS‐STING‐IFN‐I pathway also showed increased phosphorylation levels in IBA1^+^ microglia from NMO‐IgG group (Figure S6A,B, Supporting Information).

To investigate the effect of IFN‐I response on AQP4‐specific T cells, we next immunized *Aqp4* ^‐/‐^ mice with the previously reported AQP4‐specific peptides AQP4_p135‐153_ or AQP4_p201‐220_ to obtain AQP4‐specific T cells for further study.^[^ ^20^, ^21^ ^]^ First, we immunized *Aqp4* ^‐/‐^ mice with the AQP4_p135‐153_ peptide, and then obtained lymphocytes from draining lymph nodes for Th17 polarization (IL‐23, IL‐6, and the AQP4‐specific peptide), followed by passive transfer (Figure S6C, Supporting Information).^[^ ^20^ ^]^ In the Th17‐AQP4_p135‐153_ passive transfer mice, we further validated the changes of the cGAS‐STING‐IFN‐I pathway in MACS sorted microglia from spinal cord tissue during the peak of disease at 14 days (Figure S6D, Supporting Information). Next, we immunized *Aqp4^‐/‐^* mice with the AQP4_p201‐220_ peptide. Lymphocytes were obtained from the draining lymph nodes and cultured in vitro (Figure 4H). To directly explore the impact of IFN‐I on AQP4‐specific T cells, we employed the I‐A^b^‐AQP4(205‐215) tetramers.^[^ ^21^ ^]^ The results indicated that the additional administration of IFN‐I would elevate the proportion of CD4^+^ AQP4‐specific autoreactive T cells (Figure 4I). Moreover, we observed that, in comparison with the control group and the Th17 polarization condition, additional IFN‐I stimulation could increase the proportion of IFN‐γ and IL‐17A positive cells within CD4^+^ T cells (Figure 4J). Simultaneously, Th17 polarization condition would also induce an increase in the proportion of CCR5^+^ CD4^+^ and CCR6^+^ CD4^+^ T cells (Figure 4K). Moreover, when additional IFN‐I stimulation was used under Th17 polarization conditions, this would significantly result in an increase in the proportion of CCR5^+^ CD4^+^, CCR6^+^ CD4^+^, and CXCR3^+^ CD4^+^ T cells (Figure 4K).

### 2.5 *Sting* Deficiency Alleviated NMO‐IgG Associated Pathology in NMOSD

**Figure 5:**
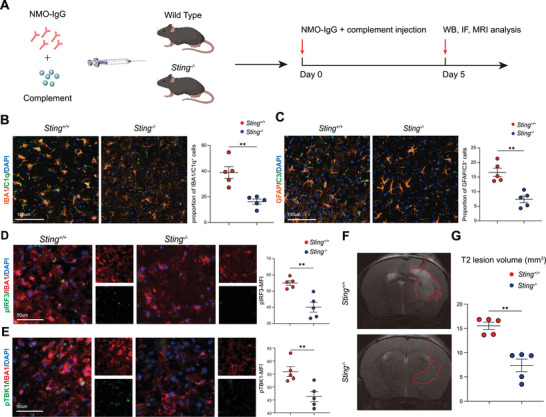
*Sting^‐/‐^* mice exhibited improved immunopathology in the NMO‐IgG model. A) Flow chart illustrating the process of NMO‐IgG intracerebral model. B) Representative immunofluorescence images and quantification of the IBA1^+^/C1q^+^ microglia around the lesion in the brain of WT mice and *Sting^‐/‐^* mice at the peak of disease (*n* = 5 per group). C) Representative immunofluorescence images and quantitative analysis showing changes in GFAP^+^/C3^+^ astrocytes around the lesion in *Sting^‐/‐^* compared to WT mice at the peak of disease (*n* = 5 per group). D) Representative immunofluorescence images and quantitative analysis of phosphorylated IRF3 (pIRF3) mean fluorescence intensity (MFI) around the lesions in WT and *Sting^‐/‐^* mice at the peak of disease (*n* = 5 per group). E) Representative immunofluorescence images and quantitative analysis of phosphorylated TBK1 (pTBK1) MFI around the lesions in WT and *Sting^‐/‐^* mice at the peak of disease (*n* = 5 per group). F) Representative MRI images showing lesion volume at the peak of the disease between WT and *Sting^‐/‐^* mice. G) MRI analysis quantifies the demyelinating lesion volume in both WT and *Sting^‐/‐^* mice at the peak of disease (*n* = 5 per group). Data were analyzed using unpaired student's t‐test or Mann‐Whitney test; Mean ± s.e.m; *p < 0.05, **p < 0.01.

We next developed *Sting* gene knockout mice and established NMO‐IgG model to investigate the role of cGAS‐STING‐IFN‐I in the pathogenesis of NMOSD (**Figure** **5**A). We first performed immunofluorescence analysis, defining cells co‐expressing IBA1 and C1q as activated microglia, and cells co‐expressing GFAP and C3 as activated astrocytes.^[^ ^4^, ^18^ ^]^ Our results revealed a significant reduction in the proportion of activated glial cells surrounding the lesion site in *Sting^‐/‐^* mice compared to *Sting^+/+^* mice (microglia: 38.9% vs 16.2%; astrocytes: 16.6% vs 7.4%) (Figure 5B,C). In addition, reduced levels of IRF3 and TBK1 phosphorylation were observed in IBA1^+^ microglia surrounding the lesion site in *Sting^‐/‐^* mice (Figure 5D,E). Moreover, using 9.4‐T MRI to visualize demyelination lesions (T2) on day 5, a significant reduction in the volume of demyelination lesions was observed in *Sting^‐/‐^* mice (17.1 vs 9.4 mm^3^) (Figure 5F,G). These findings suggest that inhibition of STING effectively suppressed the excessive activation of the IFN‐I response mediated by NMO‐IgG, which directly attacked astrocytes in NMOSD.

### 2.6 Reduction of AQP4‐Specific T Cell Inflammation and Autoreactivity by *Sting* Deficiency

**Figure 6:**
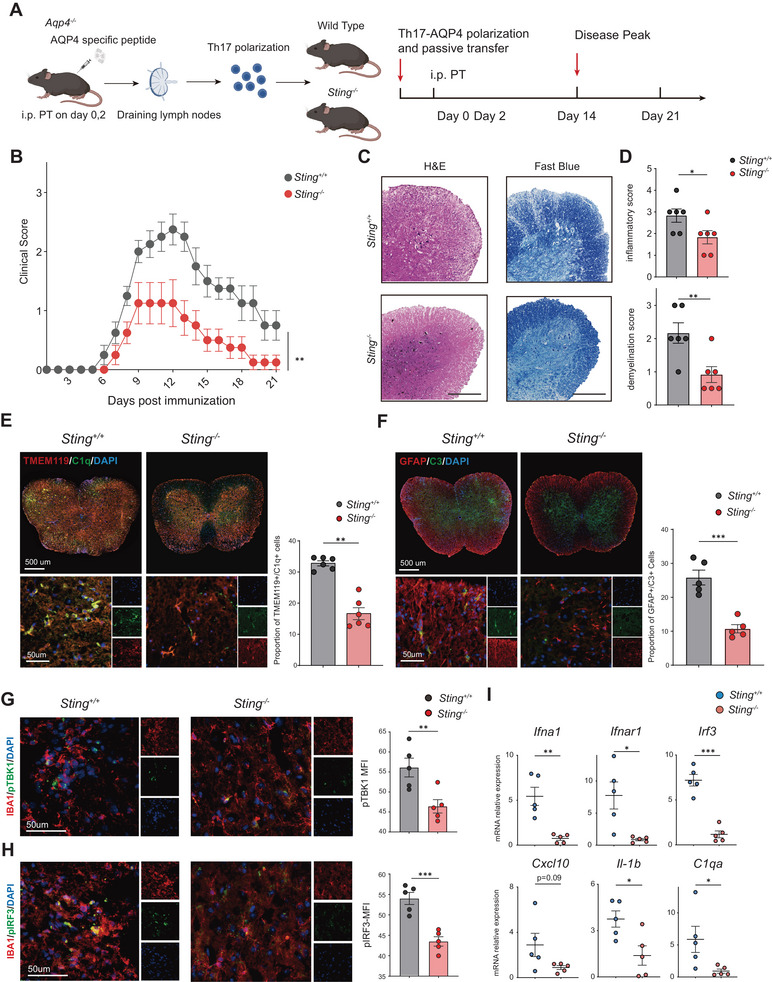
Attenuated glia damage and IFN‐I activation in AQP4 specific *Sting^‐/‐^* deficiency mouse model. A) Schematic representation of Th17‐AQP4 specific T cells passive transfer model. B) Clinical scores of the Th17‐AQP4 model were compared between WT and *Sting^‐/‐^* mice (*n* = 8 per group). C) Representative H&E and LFB staining images of Th17‐AQP4 model between WT and *Sting^‐/‐^* mice, scale=500 µm. D) Quantitative analysis revealed a significant decrease in inflammatory infiltration and demyelination level among *Sting^‐/‐^* mice (*n* = 6 per group). E) Representative immunofluorescence images and quantitative analysis were conducted to assess the presence of TMEM119^+^/C1q^+^ microglia of WT mice and *Sting^‐/‐^* mice within spinal cord lesions at the peak of the disease, indicating a significant reduction in microglial activation in *Sting^‐/‐^* mice compared to WT mice (*n* = 6 per group). F) Representative immunofluorescence images and quantitative analysis of the GFAP^+^/C3^+^ activated astrocytes in spinal cord lesions, showing lower levels of astrocyte activation in *Sting^‐/‐^* mice during the peak of the disease (*n* = 5 per group). G) Representative immunofluorescence images and quantitative analysis of pTBK1 mean fluorescence intensity were compared between IBA1^+^ microglia within spinal cord lesions of *Sting^‐/‐^* mice and WT mice (*n* = 5 per group). H) Representative immunofluorescence images and quantitative analysis of the MFI of pIRF3 were compared between IBA1^+^ microglia within the spinal cord lesions of *Sting^‐/‐^* mice and WT mice (*n* = 5 per group). I) qPCR analysis showed decreased expression of *Ifna1*, *Ifnar1*, *Irf3*, *Cxcl10*, *Il‐1b*, and *C1qa* genes in the microglia of *Sting^‐/‐^* mice during the peak of disease (*n* = 5 per group). Statistical analysis for clinical scores was performed using one‐way ANOVA with Tukey's post‐test, while unpaired student's t‐test or Mann‐Whitney test was employed for histology and flow cytometry comparisons; data presented as Mean ± s.e.m.; *p < 0.05, **p < 0.01, ***p < 0.001.

To further investigate the role of IFN‐I in the pathogenesis of NMOSD on AQP4‐specific T cells, we then used AQP4‐specific passive transfer T cell model to study the effect of the STING‐IFN‐I pathway (**Figure** **6**A). First, in the mouse model of Th17 AQP4_p135‐153_ adoptive transfer, we discovered *Sting^‐/‐^* mice exhibited faster symptom recovery with lower clinical scores in comparison to the WT (maximal mean clinical score: 2.4 vs 1.1) (Figure 6B). Histological analyses, including H&E staining and LFB staining, showed reduced inflammatory cell infiltration and demyelination in spinal cord lesions in the *Sting^‐/‐^* mice (Figure 6C,D). At peak disease, *Sting^‐/‐^* mice had less activated TEME119^+^/C1q^+^ microglia (32.8% vs 16.3%) and GFAP^+^/C3^+^ astrocytes (25.82% vs 10.72%) in their spinal cord lesions compared to WT mice (Figure 6E,F). Additionally, the phosphorylation levels of TBK1 and IRF3 were reduced in IBA1^+^ microglia within spinal cord lesions of *Sting^‐/‐^* mice (Figure 6G,H). Knockout of *Sting* also reduced IFN‐I‐associated genes in MACS‐sorted CD11b^+^ microglia during the peak disease (Figure 6I).

**Figure 7:**
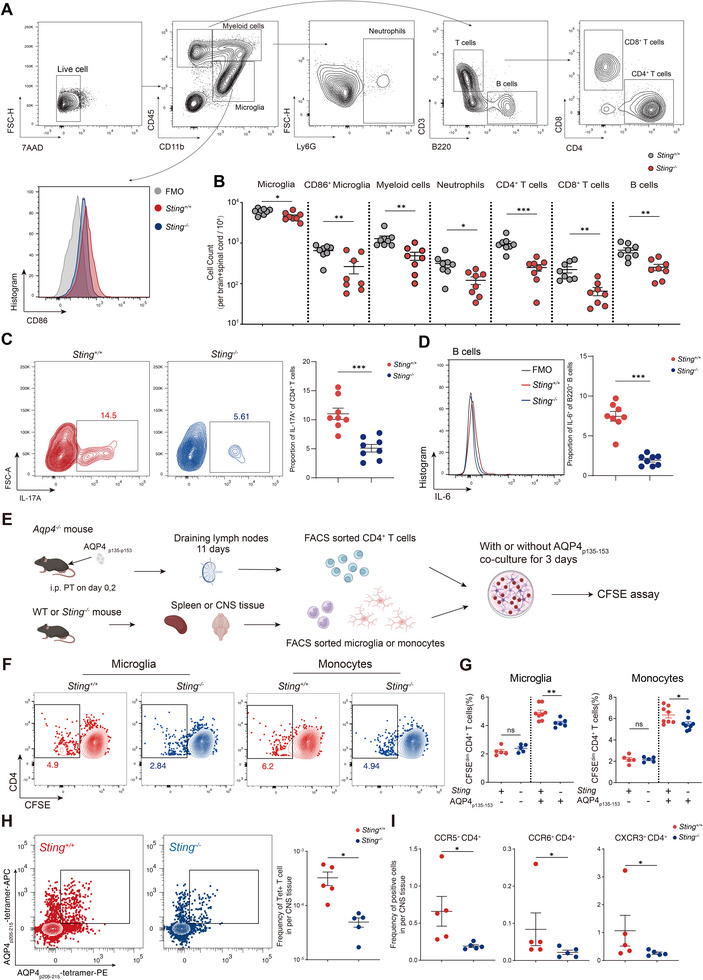
*Sting^‐/‐^* deficiency hindered the activation of AQP4‐specific T cells. A) Flow cytometry gating strategy of different cell types in the CNS of AQP4_p135‐153_ specific T cells transfer mice. B) Quantitative analysis showed a notable reduction in the proportion of the infiltrating immune cells within the CNS of WT and *Sting^‐/‐^* mice (*n* = 8 per group). C,D) The frequency of IL‐17A^+^ cells within the CD4^+^ T cells and the percentage of IL‐6^+^ cells among B cells were assessed using flow cytometry in both WT and *Sting‐/‐* mice (*n* = 8 per group). E) Flow chart depicting the process of CFSE assay in vitro. F,G) Representative flow cytometry and statistical analysis indicated that the absence of *Sting* in CNS microglia and periphery monocytes reduced the expansion of AQP4‐specific T cells in vitro (*n* = 5–8 per group). H) Representative flow cytometry and statistical analysis indicated that following the adoptive transfer of AQP4_p201‐220_ T cells, the proportion of antigen‐specific T cells in the *Sting^‐/‐^* mice was lower compared to WT mice (*n* = 5 per group). I) Flow cytometry results were analyzed to observe the alterations in the proportion of CD4^+^ chemokine receptor‐positive T cells within the CNS tissues of WT and *Sting^‐/‐^* mice following the adoptive transfer of AQP4_p201‐220_ T cells. The gating strategy was consistent with that in Figure 4K (*n* = 5 per group). Unpaired student's t‐test or Mann‐Whitney test was employed to compare results across groups; data presented as Mean ± s.e.m; *p < 0.05, **p < 0.01, ***p < 0.001.

Meanwhile, in the AQP4_p135‐153_ specific T cell adoptive transfer model, flow cytometry analysis revealed significant reductions in CD4^+^ T cells, CD8^+^ T cells, B220^+^ B cells, and Ly6G^+^ neutrophils (3.92‐, 3.39‐, 2.63‐, and 2.60‐ fold decrease, respectively) in the CNS during disease peak in *Sting^‐/‐^* mice. The population of CD45^int^CD11b^+^ microglia exhibited a 1.33‐fold decrease, accompanied by a reduction in the proportion of CD86^+^ cells within these microglia (**Figure** **7**A,B). Meanwhile, the proportions of IL‐17A‐positive cells in CD4^+^ T cells (11.03% vs 5.1%) and IL‐6‐positive cells within B cells were both significantly decreased (7.46% vs1.94%) (Figure 7C,D). While the relative proportions of T cell, B cell, and other immune cell populations remained unchanged in both the spleen and blood, there was also a significant decrease in the proportion of IL‐17A‐positive CD4^+^ T cells (0.79% compared to 0.55%) and IL‐6‐positive cells in B cells (1.07% compared to 0.61%) within the spleen (Figure S7A–D, Supporting Information). No significant changes in the proportion of T and B cells within the CNS of WT and *Sting^‐/‐^* mice were observed during the disease recovery phase (Figure S7E, Supporting Information). The co‐culture of AQP4_p135‐153_‐specific CD4^+^ T cells and microglia, sorted by FACS, also revealed that *Sting* deficiency resulted in a significant reduction in the proportion of IL‐17A positive cells (6.25% vs 4.13%) and IFN‐γ‐positive cells (4.80% vs 3.50%) within the CD4^+^ T cell population (Figure S7F–H, Supporting Information). The CFSE assay in vitro demonstrated that *Sting* deficiency in microglia and monocytes could effectively suppress the expansion of AQP4 _p135‐153_‐specific CD4^+^ T cells (Figure 7E,G). Following the adoptive transfer of AQP4_p201‐220_‐specific T cells, antigen‐specific T cells were detected in both the WT mice and *Sting^‐/‐^* mice. The results indicated that *Sting^‐/‐^* mice presented a decreased proportion of I‐A^b^‐AQP4(205‐215) tetramer positive T cells (Figure 7H). Meanwhile, the proportion of chemokine receptor CCR5^+^, CCR6^+^, and CXCR3^+^ cells within CD4^+^ T cells in the CNS of *Sting^‐/‐^* mice also decreased significantly (Figure 7I). *Sting* was knocking down in AQP4_p201‐220_ specific T cells by using retrovirus encoding shRNA. We discovered that under Th17 polarization conditions in vitro, the knockdown of *Sting* could significantly reduce the proportion of IL‐17A and IFN‐γ positive cells within CD4^+^ T cells (Figure S8A,B, Supporting Information). Following adoptive transfer of retrovirus‐transduced cells into WT mice, compared to those receiving shRNA‐NC transduced cells, mice receiving shRNA‐*Sting* transduced cells exhibited a significant reduction in the proportions of CD4⁺ T cells, CD8⁺ T cells, and B220⁺ B cells infiltrating the CNS (Figure S8C, Supporting Information). Meanwhile, the proportion of I‐A^b^‐AQP4(205‐215) tetramer positive T cells also significantly decreased (Figure S8C, Supporting Information). These findings indicate that STING inhibition could effectively attenuate disease severity and reduce activation of AQP4‐specific T cells.

### 2.7 Treatment with STING Inhibitor H‐151 Alleviated NMOSD Mouse Models

**Figure 8:**
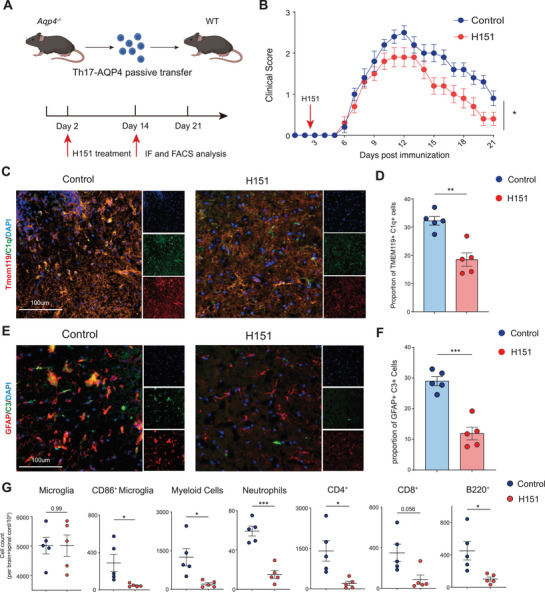
STING inhibitor H‐151 effectively improved the AQP4‐specific T cells model. A) Schematic representation of Th17‐AQP4 model treated with STING inhibitor H‐151. B) Clinical symptom scores compared between control group mice and mice treated with H‐151 in the Th17 mouse model (*n* = 8 per group). C) Representative immunofluorescence images depicting changes in TMEM119^+^/C1q^+^ microglia within the spinal cord lesion of WT mice and H‐151‐treated mice at the peak of disease. D) Quantitative analysis of TMEM119^+^/C1q^+^ microglia (*n* = 5 per group). E) Representative immunofluorescence images demonstrating alterations in GFAP^+^/C3^+^ astrocytes within the spinal cord lesion of WT mice and H‐151‐treated mice at the peak of disease. F) Quantitative analysis revealing changes in GFAP^+^/C3^+^ astrocyte (*n* = 5 per group). G) Flow cytometry analysis to quantify CNS‐infiltrated cells in both WT and H‐151‐treated mice at the peak of disease. The gating strategy was consistent with that in Figure 7A (*n* = 5 per group). Statistical analyses: Clinical scores were assessed using one‐way ANOVA with Tukey's post‐test. Unpaired student's t‐test or Mann‐Whitney test was used to compare results from flow cytometry and immunofluorescence studies. Data were presented as Mean ± s.e.m, *p < 0.05, **p < 0.01, ***p < 0.001.

To further validate the therapeutic targeting of the cGAS‐STING‐IFN‐I pathway, we employed a specific STING inhibitor, H‐151,^[^ ^22^ ^]^ to treat NMOSD in mouse models. In the Th17‐AQP4_p135‐153_ passive transfer model, mice receiving H‐151 treatment exhibited notable improvements in clinical symptom scores compared to the control group (maximal mean clinical score: 2.5 vs 1.9) (**Figure** **8**A,B). At the disease peak, immunofluorescence staining of spinal cord lesions revealed a remarkable reduction in both microglial and astrocyte activation in H‐151 treated mice compared to WT mice (Figure 8C–F). Flow cytometry analysis further confirmed a significant reduction in the infiltration of inflammatory cells, including CD4^+^ T cells, CD8^+^ T cells, B220^+^ B cells, and Ly6G^+^ neutrophils (fold decreases of 7.12, 6.01, 4.38, and 3.90, respectively) in mice treated with H‐151 (Figure 8G). It is noteworthy that, while the overall number of CD45^int^CD11b^+^ microglia remained constant in the CNS, there was a notable reduction in the percentage of CD86‐positive cells within CD45^int^CD11b^+^ microglia (Figure 8G). Additionally, in the NMO‐IgG model, mice treated with H‐151 also demonstrated a significant reduction in microglia and astrocyte activation compared to WT mice (Figure S9, Supporting Information). These findings collectively support the potential of targeting the STING pathway as a therapeutic approach for NMOSD treatment.

## 3 Discussion

Recent studies imply that a dysregulated recognition of self‐DNA through the cGAS‐STING pathway contributes to the development of autoimmune disorders.^[^ ^23^, ^24^, ^25^, ^26^ ^]^ Whether and how this pathway operates in CNS autoimmune diseases, i.e., NMOSD, remains unexplored. Previous studies have demonstrated that cfDNA derived from neutrophil extracellular trap‐related cell death (NETosis) can induce abnormal IFN‐I responses in the peripheral blood of patients with NMOSD.^[^ ^27^ ^]^ In our study, not only was an increase in cfDNA concentration detected in the plasma of NMOSD patients, but also in their CSF. Simultaneously, the leakage of mitochondrial DNA is a crucial factor in activating the cGAS‐STING. Previous studies have shown that during aging, the leakage of mitochondrial DNA in microglia leads to the activation of the cGAS‐STING pathway.^[^ ^19^ ^]^ Our findings also indicate that NMO‐IgG could cause abnormal mitochondrial damage and abnormal intracellular DNA accumulation in microglia. These results suggest that in NMOSD, extracellular cfDNA and intracellular mitochondrial DNA leakage in the CNS might jointly contribute to the activation of the cGAS‐STING pathway in microglia. After cGAS activation, cGAMP is usually released and absorbed by bystander cells via gap junctions, resulting in the activation of STING beyond the actual site of cGAS activation.^[^ ^15^, ^16^ ^]^ Consequently, elevated plasma cGAMP levels in patients with NMOSD suggest that it may function by STING‐dependent IFN‐I production.

In our scRNA‐seq subcluster analysis of myeloid cells in both the CSF and peripheral blood, we identified a specific cluster exhibiting expression of the chemokine CCL4. These CCL4^+^ microglia‐like cells and monocytes may regulate Th17 cell differentiation, as evidenced by the accompanying upregulation of IL17RA in NMOSD. The GSEA analysis identified the activation of B cell‐mediated immunity in the microglia and macrophages in the CSF, further substantiating the role of IFN‐I response in driving aberrant activation in NMOSD patients. Further, this analysis reinforces the regulatory role of IFN‐I in modulating the inflammatory responses of T cells and B cells in NMOSD patients.^[^ ^2^, ^28^, ^29^, ^30^ ^]^ Our analysis of CD4^+^ T cell subclusters in NMOSD patients reveals an augmentation of T cell inflammatory response. Pseudotime analysis indicated an elevated expression of IFN‐I response genes during T‐cell differentiation and maturation, underscoring the significant impact of IFN‐I on T cells response in the NMOSD.^[^ ^8^, ^31^ ^]^ A previous study has shown that the frequency of AQP4‐specific effector memory CD4^+^ T cells in patients with NMOSD were increased with an effector memory phenotype, and the autoreactive T cells in patients with NMOSD may undergo an exhaustion‐like phenotype under chronic stimulation. This phenotype was characterized by a gradual loss of pro‐inflammatory cytokine expression and acquisition of co‐inhibitory receptors, along with the expression of *FOXP3*. Although these cells show exhaustion features, they still retain the ability of B cell helper function.^[^ ^32^ ^]^ In our study, IFN‐I pathway related genes showed increased expression when T cells were differentiated into the terminal stage. During in vitro culture of AQP4‐specific T cells, we found that IFN‐I increased the proportion of AQP4 antigen‐specific T cells. However, the AQP4‐specific T cells of patients with NMOSD did not show an abnormal IFN‐I response in a previous study.^[^ ^32^ ^]^ We speculate that the difference may be due to the fact that some of the patients in that study were in the period of remission of NMOSD. In contrast, all the enrolled patients in our study were in the acute phase. During the disease active stage, IFN‐I promotes the presentation of AQP4 antigen by antigen‐presenting cells by enhancing the expression of MHC‐II molecules,^[^ ^33^ ^]^ accelerating the activation and clonal expansion of autoreactive T cells, which may be a key mechanism in the acute stage of the disease. However, long‐term exposure to IFN‐I may induce the expression of co‐inhibitory receptors (such as *TIM‐3* and *LAG‐3*) in T cells. This dynamic balance may manifest as the “self‐limiting” property of IFN‐I signaling.^[^ ^34^ ^]^ Meanwhile, we found that the additional use of IFN‐I under the Th17 polarization conditions (IL‐6 and IL‐23) could simultaneously increase the proportion of IL‐17A and IFN‐γ positive T cells. This finding reminiscent a previous study which also showed that in the Th17‐EAE model, the additional treatment with IFN‐β leads to an increase in GM‐CSF and IL‐17 positive T cells in mice.^[^ ^8^ ^]^ Given that the treatment of IFN‐I has an immunomodulatory effect of inhibiting Th1 cells in MS,^[^ ^35^ ^]^ our study further reveals the dual role of IFN‐I in regulating T cell responses under complex autoimmune states.

Here, we provide molecular evidence suggesting that intrinsic activation of the myeloid cell IFN‐I response is implicated in NMOSD exacerbation. In contrast to the worsening effects of IFNβ‐1b, blocking the cGAS‐STING‐IFN‐I pathway can mitigate NMO‐IgG‐induced CNS pathogenicity and reduce the activation of AQP4‐specific T cells. Our research demonstrated that *Sting* deficiency significantly improved clinical symptoms in NMOSD mouse models by reducing the activation of microglia and astrocytes, as well as AQP4‐specific T cells. This is consistent with previous studies that demonstrated a correlation between aberrant activation of the IFN‐I pathway and microglia in mouse models and *Ifnar* knockout alleviated pathological changes in NMOSD.^[^ ^36^ ^]^ Furthermore, a recent study using AQP4_p201‐220_ induced mouse model showed that *Ifnar* ^‐/‐^ mice had a lower incidence and clinical score.^[^ ^37^ ^]^

Anifrolumab, a monoclonal antibody targeting IFNAR, has been approved for treating systemic lupus erythematosus due to its effective inhibition of the IFN‐I activity.^[^ ^38^, ^39^ ^]^ Currently, a range of specific pharmaceuticals targeting cGAS or STING are also under development as potential clinical candidates for the treatment of autoimmune disorders.^[^ ^40^ ^]^ For instance, H‐151, serving as an effective STING inhibitor in both human and murine models, exhibits significant suppression of systemic cytokine response through reduction of IFN‐I reaction.^[^ ^22^ ^]^ Hence, for NMOSD patients with aberrant activation of the cGAS‐STING‐IFN‐I pathway, targeting this pathway may offer novel therapeutic options that could concurrently influence myeloid cells and self‐reactive T and B cells. These findings warrant further investigation to assess the potential of such therapies in the treatment landscape of NMOSD.

## 4 Experimental Section

### Process of scRNA‐Seq Sample Acquisition

Five AQP4‐IgG seropositive NMOSD patients were enrolled from Tianjin Medical University General Hospital. All participants fulfilled the 2015 criteria for NMOSD as established by the International Panel for NMO Diagnosis (IPND).^[^ ^41^ ^]^ Clinical information was obtained from the medical records of hospitalized patients. Before participation, all individuals provided informed consent. Full informed consent was acquired and the work was carried out under the approvals of the Research ethics committee IRB2021‐KY‐134. Additionally, paired CSF and PBMCs samples were collected from 3 healthy controls and 5 control non‐neuroinflammatory samples from the GEO database (GSE133028, GSE138266) to serve as control groups for subsequent analysis. The demographic characteristics of patients and controls are detailed in Table S1 (Supporting Information). For each newly enrolled patient with NMOSD, 5 ml of peripheral blood and 10 ml of CSF samples were collected for subsequent experimental analysis. Whole blood and CSF samples were processed similarly to the previously described methodology.^[^ ^9^, ^10^, ^11^ ^]^ CSF cells were obtained via centrifugation, and PBMCs were isolated from fresh whole blood using the Ficoll‐Paque density gradient method. One CSF sample could not be used for subsequent experiments due to low cell activity. For scRNA‐seq library preparation, 5′ library preparation kits from 10x Genomics (Pleasanton, CA, USA) were utilized.

### scRNA‐seq Data Analysis

Due to the incorporation of scRNA‐seq samples from various studies into the integrated dataset, data quality control was initially conducted on the expression matrices of all included samples. In order to address batch effects and harmonize the data from all samples, the canonical correlation analysis (CCA) algorithm in Seurat (v4.3.0.1) was employed to mitigate confounding factors such as different sequencing platforms and tissues. The typical UMAP plots before and after data integration are presented in Figure S1B (Supporting Information). Cell clustering was performed with the graph‐based clustering algorithm in the *FindClusters* function, set at a resolution of 0.6. UMAP visualization was used to display the identified clusters. The Wilcoxon test was utilized for differential gene analysis using the *FindAllMarkers* function. Additionally, covariates such as age and gender were incorporated into the analysis process to mitigate batch effects, with a log fold change threshold set at 0.25. Enriched pathways were evaluated through hypergeometric testing in both Gene Ontology (GO) and Kyoto Encyclopedia of Genes and Genomes (KEGG) databases. The *compareCluster* function in the clusterProfiler package was used for pathway enrichment analysis. Pathways with a Benjamini–Hochberg corrected p‐value < 0.05 were considered significantly enriched. To illustrate possible differential activity among cell groups and disease conditions, Gene Set Enrichment Analysis (GSEA) score calculation was performed for monocyte and microglia‐like cell sub‐clusters. Hallmark gene sets were obtained from MSigDB, excluding overlapping genes within each gene set. The AUCell package (V1.12.0)^[^ ^42^ ^]^ was used to calculate the signature score of a specific gene set. First the ranked expression matrix was built using the *AUCell_buildRankings* function, and then calculated the AUC value using the *AUCell_calcAUC* function. The genes in the utilized gene sets are shown in Table S4 (Supporting Information). The cGAS‐STING‐ IFN‐I gene set was obtained from the relevant pathway genes in the MSigDB database. Gene sets associated with Th1, Th2, Th17, T cell activation, proliferation, and cytotoxicity, were compiled based on previous research.^[^ ^43^ ^]^ The Monocle package (V2.18.0) was used to reconstruct the cellular differentiation trajectory of CD4^+^ T cell subsets. The naive T cell cluster (C1_CCR7) was set as roots of the trajectory to order the cells in pseudotime by using *orderCells* function. The *plot_pseudotime_heatmap* and *plot_genes_in_pseudotime* function were used to visualize the dynamic expression changes of significantly DEGs identified by the *differentialGeneTest* function across the pseudotime of each trajectory. Subsequently, the *AUCell_calcAUC* function was employed to calculate gene‐set scores along each identified trajectory. To confirm the trajectory results from Monocle, Slingshot (V2.10.0) was applied to infer the differentiation trajectory.

### CSF and Plasma cfDNA Analysis

Cell‐free double strand DNA was detected in paired CSF and plasma samples from 20 NMOSD patients in the acute phase (first diagnosed or relapse), and 12 age and sex matched control subjects. cfDNA was analyzed using the Quant‐iT™ dsDNA (Thermo Scientific) in accordance with manufacturer's instructions.

### Plasma 2′3′‐cGAMP Detection by ELISA

Plasma samples were obtained from 36 patients diagnosed with NMOSD, as well as from 21 control subjects, for the purpose of measuring the levels of 2′‐3′ cGAMP. Among them, 32 samples were from the same subjects as those used for the above‐mentioned detection of plasma cfDNA. The concentration of 2′‐3′ cGAMP was determined using enzyme‐linked immunosorbent assays (ELISA), per the manufacturer's instructions (Invitrogen, EIAGAMP).

### Animals

*Sting^‐/‐^* mice (Strain NO. T012747) and *Aqp4^‐/‐^* mice (Strain NO. T011901) were obtained from GemPharmatech (Nanjing, China). Wild‐type female C57BL/6 mice, aged 8‐10 weeks old, were purchased from Vital River (Beijing, China). All the procedures of animal experiments had received approval from the Animal Experimental Ethics Committee of Tianjin Medical University (IRB2021‐DWFL‐216) and were conducted following the Revised Guide for the Care and Use of Laboratory Animals.

### NMO‐IgG Preparation and Intracerebral Injection Model Induction

The intracerebral NMO‐IgG injection mice model was established following methodologies from previous studies.^[^ ^17^ ^]^ The isolation of NMO‐IgG from NMO patients’ serum followed a previously described methodology. Both NMO‐IgG and Control human‐IgG (Solarbio, Beijing, China) were in a 10 mg ml^−1^ concentration in phosphate‐buffered saline (PBS). Mice were anesthetized and mounted onto a stereotactic frame (RWD Life Science, Shenzhen, Guangdong, China). A burr hole was drilled in the skull 2.5 mm to the right of the bregma under a midline scalp incision. A 30‐gauge needle attached to a 50 ul gas‐tight glass syringe (Hamilton, Reno, NV, USA) was inserted 3 mm deep to infuse 12 ul mixture of NMO‐IgG or human‐IgG supplemented with human complement (hC) (volume ratio 3:2). Mice in the STING inhibitor‐treated group received daily H151 (750 nmol; 200ul; i.p.) treatment.

### AQP4‐Specific T Cells Mouse Model Induction

The AQP4‐specific T cells mouse model was established following methodologies from previous studies.^[^ ^20^, ^44^ ^]^ C57BL/6 *Aqp4^‐/‐^* mice were immunized with 200 µg of AQP4 peptides p135‐153 or p201‐220 (GeneScript Synthesis) in Freund's complete adjuvants containing Mycobacterium tuberculosis H37Ra. Meanwhile, mice received intraperitoneal injection of 200 ng pertussis toxin (Ptx) (List Biological) on days 0 and 2 after immunization. Draining lymph nodes were dissected on day 10‐12 after immunization. Th17 polarizing conditions were cultured with corresponding AQP4 peptides (AQP4_p135‐153_ or AQP4_p201‐220_) (10 µg ml^−1^), IL‐23 (20 ng ml^−1^; R&D Systems), and IL‐6 (20 ng ml^−1^; R&D Systems) in complete RPMI 1640 medium (Gibco) for three days. Donor mice were intravenously injected with 1.5‐2 × 10^7^ cells per mouse, and PT solution was i.p. injected on the day of induction and 48 h later. Clinical monitoring included daily assessments of body weight and symptoms post‐model induction. Paralysis severity was scored using a canonical clinical scale: 0) healthy; 0.5) partial tail weakness; 1) complete loss of tail tone; 2) partial hind‐limb paralysis; 2.5) Paralysis of one hind limb; 3) complete hindlimb paralysis; 4) forelimb paralysis; and 5) moribund/dead. Mice in the STING inhibitor‐treated group received daily treatments of H151 (750 nmol; 200ul; i.p.).

### Bulk RNA Sequencing Analysis

Brain tissues were enzymatically digested following established protocols.^[^ ^45^ ^]^ Following model induction, mice were euthanized humanely, and brain tissues were collected and processed. Tissues were minced and incubated with collagenase IV and deoxyribonuclease at 37 °C for 30 min. After removing myelin debris through centrifugation using 30% Percoll, single cells were suspended in PBS supplemented with 2% FBS. Microglia isolation from the NMO‐IgG injection mouse model was achieved by utilizing flow cytometry sorting (FACS Aria III, BD Biosciences, San Jose, CA, USA). Libraries prepared from these samples underwent sequencing on an Illumina NovaSeq platform.^[^ ^17^ ^]^ Count data were generated using featureCounts, and the expression profiles of cGAS‐STING‐IFN‐I, disease‐associated microglia, and chemokine genes were normalized and visualized through the heat map.

### Transmission Electron Microscopy (TEM)

Transmission electron microscopy (TEM) samples were processed as described previously.^[^ ^17^ ^]^ Briefly, perfusion sampling was carried out 5 days after the induction of the NMO‐IgG model. First, 20 ml of PBS was perfused, followed by 30 ml of fixative (2.5% glutaraldehyde, 4% paraformaldehyde in phosphate buffer; 0.1 M). Then, it was rinsed with phosphate buffer PBS and kept overnight in PBS, followed by fixation with 2% osmium tetroxide. The samples fixed with osmium tetroxide were dehydrated and embedded in epoxy resin. Ultra‐thin sections were stained with uranyl acetate and lead citrate, and the samples were examined using a Hitachi HT7800 transmission electron microscope at an accelerating voltage of 60 kV. Damaged mitochondria were defined as those having lost cristae and/or disrupted outer and/or inner mitochondrial membranes.^[^ ^19^ ^]^ The number of damaged mitochondria was manually counted using ImageJ software.

### Immunofluorescence Staining

Brain and spinal cord tissues were fixed in OCT compound (Tissue‐Tek, Miles) and sectioned at 8um (Leica CM1850, Leica Instruments) for immunofluorescence study. The sections were incubated with following primary antibodies: rabbit anti‐TMEM119 antibody (1: 500, Abcam, ab209064), mouse anti‐GFAP antibody (1: 1000, Abcam, ab4648), goat anti‐IBA1 antibody (1: 1000, Abcam, ab5076), rat anti‐C3 antibody (1: 200, Abcam, ab11862), mouse anti‐C1q antibody (1: 200, Abcam, ab71940), mouse anti‐DNA antibody (1:200, Progen, AC‐30‐10), rabbit anti‐pIRF3 antibody (1: 100, CST, 29 047), rabbit anti‐pTBK1 antibody (1: 100, CST, D52C2). TMEM119 and IBA1 were utilized for the identification of microglia, whereas GFAP was employed to label astrocytes. After overnight incubation at 4 °C, sections were washed and exposed to the appropriate corresponding secondary antibodies. Three to five corresponding sections were utilized for staining per animal, and fluorescence signal detection was conducted using the Leica TCS SP5 laser scanning. The data were analyzed quantitatively using the ImageJ software. The cell count proportion was determined by the proportion of targeted cells within the lesion site at 20× magnification relative to the total number of cells in that area. Meanwhile, for assessing the DNA accumulation and phosphorylation level of the IRF3 or TBK1 in microglia, the mean fluorescence intensity (MFI) value of IBA1^+^ cells within a region of interest (ROI) of the same size was calculated at 40× magnification. Mean values from different sections of each animal were used for statistical analysis of differences.

### Histology Staining

The spinal cord sections were prepared as described for the immunofluorescence staining methodology. Hematoxylin & Eosin (H&E) staining of spinal cord sections was conducted to visualize inflammatory infiltration (Beijing Solarbio Science & Technology Co, G1121), and Luxol Fast blue (LFB) staining (Abcam, ab150675) was performed to visualize the demyelination of the spinal cord. Staining was conducted according to the manufacturer's protocols. Inflammation and demyelination scores were quantified from three different spinal cord sections as previously reported.^[^ ^46^, ^47^ ^]^

### Quantitative PCR Analysis

Brain and spinal cord tissues were enzymatically digested as previously described, and CD11b^+^ cells were isolated by incubating the single‐cell suspension with PE anti‐CD11b antibody (Biolegend, M1/70) and subsequently with anti‐PE microbeads (Miltenyi, 130‐048‐801). After sorting CD11b^+^ cells, reverse transcription (RT) was performed using EZ‐press Cell to cDNA Kit PLUS (EZBioscience), according to the protocol provided by the manufacturer. Gene expression was detected by real‐time PCR with FastStart Universal SYBR Green Master (Roche) on CFX connect (Bio‐Rad). The primers are listed in Table S3 (Supporting Information). Relative expression level was normalized by β‐actin as a housekeeping gene and calculated by the 2_ΔΔCt method. The data were presented as fold change relative to control samples.

### Magnetic Resonance Imaging Scanning

The lesion volumes of the NMO‐IgG intracerebral injection model were evaluated using a 9.4‐T small‐animal MRI scanner. T2‐weighted images were obtained for lesion detection with the following parameters: a repetition time (TR) of 2500 ms, an effective echo time (TE) of 33 ms, a resolution of 0.078 × 0.078 mm, two signal averages, and an imaging duration of 2 min and 40 s. Twenty‐four slices with a thickness measuring 0.5 mm were obtained using a field of view measuring 2.0 cm × 2.0 cm and a matrix size of 256 × 256. The MRI data analysis was performed utilizing ImageJ and Radiant DICOM Viewer software.

### Flow Cytometry and Tetramer Analysis

Single‐cell suspensions from mouse CNS, lymph node, or spleen tissues were prepared as described previously. Spleens were processed through a 70‐µm cell strainer, and red blood cells were lysed using ACK lysis buffer. In the case of blood samples, red blood cells were lysed by adding ACK lysis buffer at room temperature for 15 min. Following centrifugation and PBS washing, cells were resuspended in a solution containing 1% bovine serum albumin (BSA) for subsequent antibody staining. In the intracellular cytokine staining experiment, a cell stimulation cocktail and protein transport inhibitor (eBioscience, 00‐4975‐93) was employed to stimulate the cells, followed by the addition of corresponding antibodies for incubation under light‐shielded conditions. The following antibodies were utilized in the staining process: anti‐mouse CD45 antibody (Clone: 30‐F11), anti‐mouse/human CD11b antibody (Clone: M1/70), anti‐mouse Ly‐6G antibody (Clone: 1A8), anti‐mouse Ly‐6C antibody (Clone: HK1.4), anti‐mouse CD86 antibody (Clone: GL‐1), anti‐mouse CD3 antibody (Clone: 17A2), anti‐mouse CD4 antibody (Clone: GK1.5), CD8a antibody (Clone: 53‐6.7), anti‐mouse/human CD45R/B220 antibody (Clone: RA3‐6B2), anti‐mouse IFN‐γ antibody (Clone: XMG1.2,), anti‐mouse IL‐17A antibody (Clone: TC11‐18H10.1), Anti‐mouse IL‐6 antibody (Clone: MP5‐20F3), and 7‐AAD Viability staining Solution (420 404). Antibodies were conjugated to fluorescent tags, including FITC, PE, PerCP‐Cy5.5, allophycocyanin (APC), PE/Cyanine7, APC/Cyanine7, and Brilliant Violet 421 (BV421). All antibodies were purchased from BioLegend. The AQP4 antigen‐specific T cells were detected by tetramer staining. The I‐A^b^‐AQP4(205‐215) tetramer was used. Briefly, the single‐cell suspensions of the CNS tissues were stained with the I‐A^b^‐AQP4(205‐215) tetramer at room temperature for 2 h, then washed with PBS, and anti‐mouse CD3 and CD4 antibodies were added and incubated at 4 °C for 30 min. Flow cytometry assays were performed using BD FACS Aria III. The definition of different immune cell subsets in mice was described as follows: CD4^+^ T cell: CD45^hi^, CD11b^‐^, CD3^+^, CD4^+^; CD8^+^T cells: CD45^hi^, CD11b^‐^, CD3^+^, CD8^+^; B cells: CD45^hi^, CD11b^‐^, CD3^‐^, B220^+^; microglia: CD45^int^, CD11b^+^; myeloid cells: CD45^hi^, CD11b^hi^; neutrophils: CD45^hi^, CD11b^hi^, Ly6g^+^. During the statistical analysis of various immune cell types in the CNS, experiments were conducted by mixing brain and spinal cord tissues from each mouse together to perform relative cell counting statistics for each specific cell type within a population of 10 000 live cells. The proportion of IL‐17A positive cells among CD4^+^ T cells and the proportion of IL‐6 positive cells within B cells were quantified, and the gating strategy for cytokine experiments was established using fluorescence minus one (FMO) control. For the analysis of AQP4 antigen‐specific T cells, only T cells that are double positive for anti‐PE and anti‐APC fluorescence are identified as antigen‐specific T cells, and the proportion of these cells within all living cells was calculated. Flowjo 10.6.2 software was used for data analysis.

### In Vitro IFN‐I Stimulation of AQP4‐Specific T Cells Assay

Ten days after immunizing *Aqp4^‐/‐^* mice with AQP4_p201‐220_, the draining lymph nodes were acquired and processed to obtain lymphocytes. The lymphocytes were then resuspended in complete RPMI‐1640 medium and seeded at a density of 3 × 10^5^/200 µl in 96‐well plates pre‐coated with anti‐CD3 and anti‐CD28 antibodies (5 µg ml^−1^ each, all from BioLegend, clones 145‐2C11, 37.51). The cells were cultured under three conditions: The culture groups comprised the control group: Baseline culture with CD3/CD28 co‐stimulation alone, Th17 polarization conditions: AQP4_p201‐220_, 10 µg ml^−1^; IL‐23, 20 ng ml^−1^; and IL‐6, 20 ng ml^−1^, and Th17 polarization conditions with additional IFN‐I stimulation: IFNA1 (MCE, HY‐P700183AF), IFN‐β (MCE, HY‐P73130), both at 100 U ml^−1^. After 3 days of in vitro culture, the proportions of chemokine receptor CXCR3^+^, CCR5^+^, CCR6^+^ cells and IL‐17A^+^, IFN‐γ^+^ cells in CD4^+^ T cells were detected by flow cytometry. For the detection of antigen‐specific T cells, I‐A^b^‐AQP4(205‐215) tetramer was employed for flow cytometry following 5 days of in vitro culture. Cells were treated with 0.7 U/ml neuraminidase (Sigma‐Aldrich, N3001) and 10 nm dasatinib (Selleck) at 37 °C and 5% CO_2_ for 30 min prior to staining, and subsequently stained with I‐A^b^ tetramers for 2 h at room temperature with repeated resuspension. Data analysis was performed using Flowjo 10.6.2.

### Retroviral Transduction, and Adoptive Transfer

AQP4_p201‐220_‐specific T cells were isolated from immunized *Aqp4^‐/‐^* mice as previously described. Subsequently, the lymphocytes were activated with anti‐CD3 and anti‐CD28 antibodies (5 µg ml^−1^ each) in complete RPMI 1640 medium for 48 h. Activated lymphocytes were transduced with retroviruses encoding either green fluorescent protein (GFP) with scramble shRNA (shRNA‐negative control), or GFP with mouse *Sting*‐targeting shRNA for 72 h. Transduced lymphocytes were then polarized under Th17 conditions (10 µg ml^−1^ AQP4_p201‐220_, 20 ng ml^−1^ IL‐6, and 20 ng ml^−1^ IL‐23) for 3 days. For the cells utilized for adoptive transfer, after two washes, they were adoptively transferred into recipient WT mice through the vein at a dose of 1 × 10^7^ cells per mouse. PT was administered intraperitoneally at transfer and 48 h post‐transfer. Meanwhile, for the cells directly employed for detection after in vitro culture, they were processed using the flow cytometry method previously described.

### Primary Microglia and CD4^+^ AQP4‐Specific T Cells Co‐Culture Assay

Primary microglia were isolated from WT or *Sting^‐/‐^* mice brain tissue. The CNS tissues were surgically extracted and incubated with collagenase IV at 37 °C for 30 min. Following myelin removal using 30% Percoll gradient, microglia (CD45^int^CD11b^+^) isolation was achieved through FACS. CD4^+^ T cells were FCAS sorted from AQP4_p135‐153_ immunized *Aqp4^‐/‐^* mice. Primary microglia (5 × 10^4^/50 µl) were co‐cultured with AQP4‐specific T cells (1 × 10^5^/200 µl) per well with or without corresponding AQP4_p135‐153_ (20 µg ml^−1^) stimulation at 37 °C and 5% CO_2_ for 3 days. Flow cytometry was employed for the quantitative assessment of IL‐17A and IFN‐γ positive cell proportions within CD4^+^ T cells, as previously outlined. Data analysis was performed using Flowjo 10.6.2.

### Carboxyfluorescein Succinimidyl Ester (CFSE) Assay

CD4^+^ T cells were sorted using FACS from AQP4_p135‐153_ immunized *Aqp4^‐/‐^* mice, as previously described. Primary microglia (CD45^int^CD11b^+^) and monocytes (CD45^+^CD11b^+^Ly6c^+^) from CNS or spleen were aseptically sorted by FACS from WT or *Sting^‐/‐^* mice. The CD4^+^ T cells (10 × 10^6^ cells ml^−1^) suspensions were prepared and incubated with 0.5 µm CFSE away from light at room temperature for 5 min. Then, CFSE labeling was stopped by adding a 5× excess volume of cold complete RPMI medium. Cells labeled with CFSE were seeded at 1 × 10^5^/200 µl per well in 96‐well plates and the above primary microglia or monocytes were co‐cultured with the CFSE‐labelled CD4^+^ T cells at 5 × 10^4^/50 µl (at a ratio of 1:2) per well with or without AQP4_p135‐153_ (20 µg ml^−1^) stimulation at 37 °C and 5% CO2 for 3 days. CFSE‐labeled cells were collected and washed with PBS, and cells were resuspended in 1% BSA/PBS and stained with anti‐mouse CD3 and CD4 antibodies and 7‐AAD at 4 °C in the dark for 30 min. Flow cytometry was performed, and data were analyzed using FlowJo 10.6.2 software.

### Statistical Analysis

The experimental data were presented as Mean ± s.e.m. Statistical comparisons between two groups were performed using an unpaired Student's t‐test, with the Mann‐Whitney test applied for non‐parametric data. ANOVA with Tukey's post‐test was used for multiple group comparisons, and the Kruskal‐Wallis test with Dunn's post‐test was used for non‐parametric data. Statistical significance was determined at the following p‐values: *P < 0.05; **P < 0.01; ***P < 0.001. All experimental data analysis and statistical evaluation were performed using GraphPad Prism 9 software.

## Conflict of Interest

The authors declare no conflict of interest.

## Author Contributions

T.‐X.Z., X.Y., and X.G. contributed equally to this work and are co‐first authors. C.Z., F.‐D.S., A.L., and Z.K. formulated and designed the study. T.‐X.Z., X.Y., X.G., X.D., Y.L., and Z.H. performed the experiments. T.‐X.Z., X.G., X.D., X.L., N. S., Y.L., Z.H., and D.J. were involved in data collection, T.‐X.Z., X.Y., X.G., Z.L., and C.Z. analyzed all the results. T.‐X,Z., F.‐D.S., and C.Z. wrote the manuscript. All authors critically reviewed the manuscript.

## Supporting information



Supporting Information

## Data Availability

The data that support the findings of this study are available from the corresponding author upon reasonable request.
