# Supplementary material for: Type I Interferon Signaling Augments Autoimmunity in Neuromyelitis Optica Spectrum Disorder
Source: Adv Sci (Weinh). 2025 Jul 21;12(38):e00942. doi: 10.1002/advs.202500942 (PMC12520554; doi:10.1002/advs.202500942)
Supplement: Supplementary file 1 — Supporting Information [file ADVS-12-e00942-s001.pdf]

ADVANCED SCIENCE
Open Access

Supporting Information

for *Adv. Sci.*, DOI 10.1002/advs.202500942

Type I Interferon Signaling Augments Autoimmunity in Neuromyelitis Optica Spectrum Disorder

Tian-Xiang Zhang, Xiaoxiao Yang, Xue Gao, Xiaoshan Du, Xuegan Lian, Naiyuan Shao, Ye Liu, Zhenning Huang, Dongmei Jia, Alexander Y. L. Lau, Zhiguo Li, Zaal Kokaia, Fu-Dong Shi and Chao Zhang**

Supporting Information for

Type I interferon signaling augments autoimmunity in neuromyelitis optica spectrum disorder

Authors: Tian-Xiang Zhang[1, †], Xiaoxiao Yang[2, †], Xue Gao[1, †], Xiaoshan Du[1], Xuegan Lian[3], Naiyuan Shao[4], Ye Liu[1], Zhenning Huang[1], Dongmei Jia[1], Alexander Y. L. Lau[5], Zhiguo Li[1], Zaal Kokaia[6], Fu-Dong Shi[1,7]*, Chao Zhang[1,3,7, 8]*

Affiliations:

[1] Department of Neurology, Tianjin Medical University General Hospital, Tianjin, China.

[2] Department of Bioinformatics, School of Basic Medical Sciences, Tianjin Medical University, Tianjin, China.

[3] Department of Neurology, The Third Affiliated Hospital of Soochow University, Changzhou, Jiangsu, China.

[4] Department of Neurosurgery, The Third Affiliated Hospital of Soochow University, Changzhou, Jiangsu, China.

[5] Division of Neurology, Department of Medicine and Therapeutics, Faculty of Medicine, The Chinese University of Hong Kong, Shatin, Hong Kong, China.

[6] Laboratory of Stem Cells and Restorative Neurology, Lund Stem Cell Center, Lund University, SE-22184 Lund, Sweden.

[7] Department of Neurology, China National Clinical Research Center for Neurological Diseases, Beijing Tiantan Hospital, Capital Medical University, Beijing, China.

[8] State Key Laboratory of Experimental Hematology, National Clinical Research Center for Blood Diseases, Haihe Laboratory of Cell Ecosystem, Tianjin, China.

[†] Tian-Xiang Zhang, Xiaoxiao Yang, and Xue Gao have contributed equally to this research.

***Corresponding Author:** chaozhang@tmu.edu.cn, fshi@tmu.edu.cn, Department of Neurology, Tianjin Medical University General Hospital, Tianjin, China.

The PDF file includes:

Figure S1 to S9,

Tables. S1 to S4.

Supplementary figure

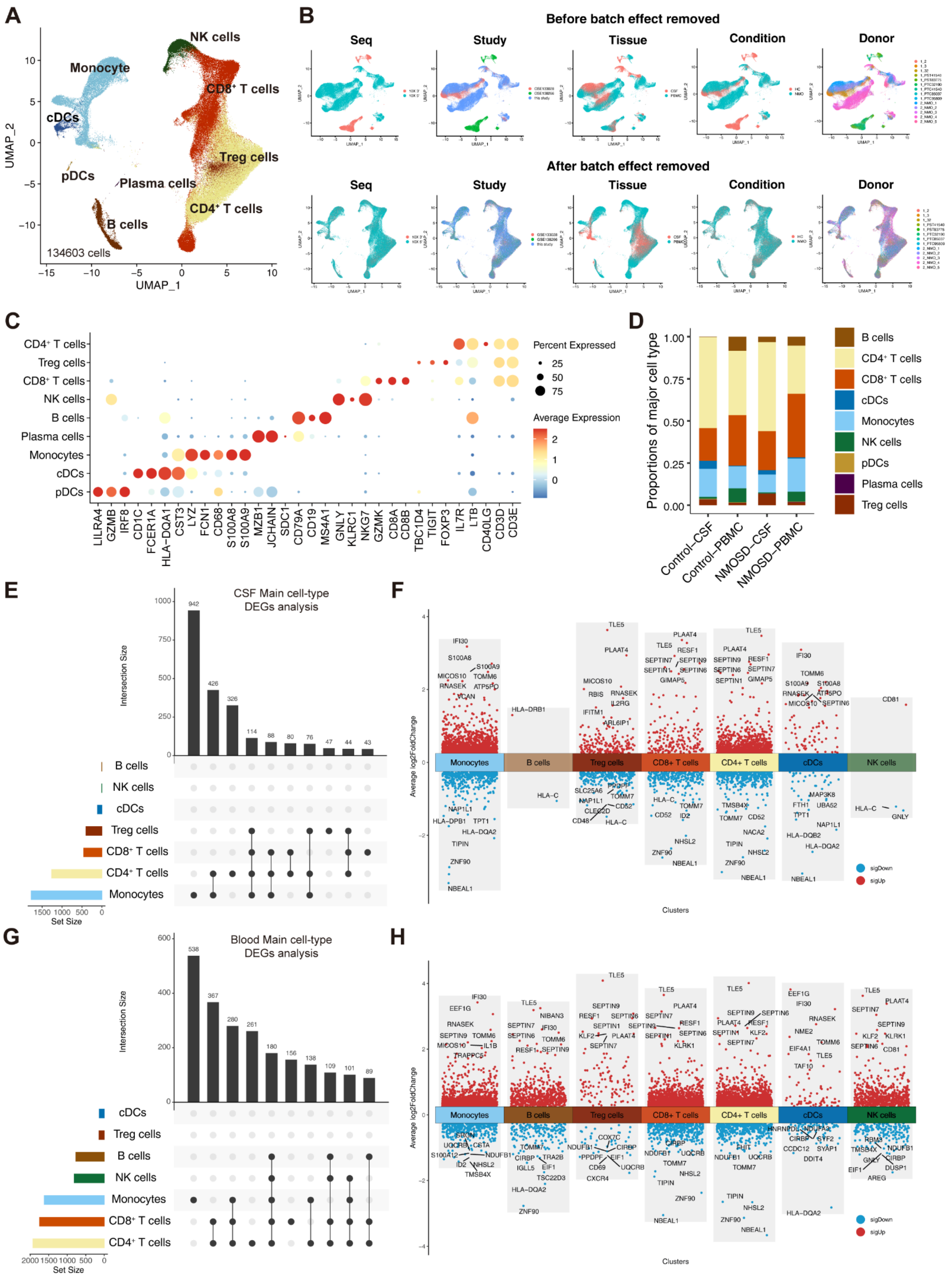

Figure S1. Single-cell RNA sequencing revealed myeloid cells and T cells with more pronounced transcriptomic alterations in peripheral blood and CSF in NMOSD.

A. UMAP plot depicting the main cell clusters identified from scRNA-seq data. B. The UMAP plot shows the effect before and after data integration and batch effect removal, revealing no significant differences between samples from distinct studies post-integration, and there is tissue heterogeneity in monocytes derived from CSF and peripheral blood. C. Dot plot illustrating the marker genes employed for classifying the main cell clusters. D. Proportions of main cell clusters observed in peripheral blood and CSF samples from both control subjects and NMOSD patients. E. UpSet plot illustrated the differential expression of genes (DEGs) across distinct cell clusters in the CSF, highlighting significant alterations predominantly in monocytes and T cells clusters. F. Volcano plots depicting DEGs of the most altered cell clusters in the CSF. G. UpSet plot illustrated monocytes and T cells clusters exhibiting a larger size of DEGs within the main cell clusters in peripheral blood. H. Volcano plots depicting DEGs of the most altered cell clusters in the peripheral blood.

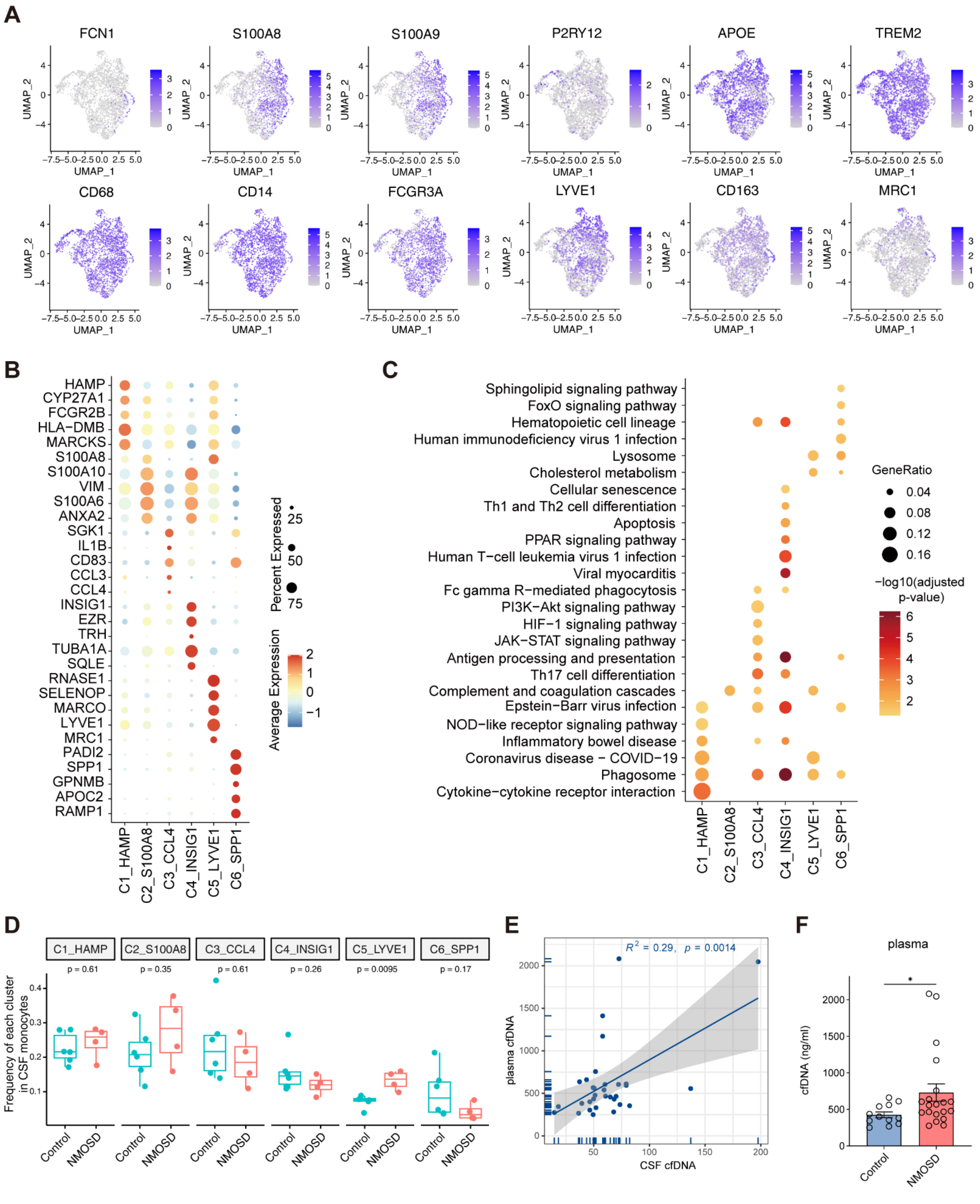

Figure S2. IFN-I displayed aberrations in the CSF monocytes and microglia.

A. UMAP plot showed the expression of classical monocyte, microglia, and macrophage markers in the CSF monocytes, microglia-like cells, and CNS-associated macrophages. B. Dot plot depicting highly expressed

marker genes specific to each sub-cluster. C. Dot plot displaying representative KEGG pathways enriched by the highly expressed genes in CSF monocyte, CNS-associated macrophages, and microglia sub-clusters. D. Boxplot illustrating differences in the frequency of CSF monocytes sub-clusters between NMOSD patients and controls. E. The correlation analysis of cfDNA levels in plasma and CSF indicated that there was a positive correlation between the cfDNA levels in the peripheral blood and CSF. F. The levels of cfDNA in plasma were detected from both Control and patients with NMOSD (Control = 12, NMOSD = 20). Data were analyzed using Mann-Whitney test; Mean ± s.e.m; * $p < 0.05$.

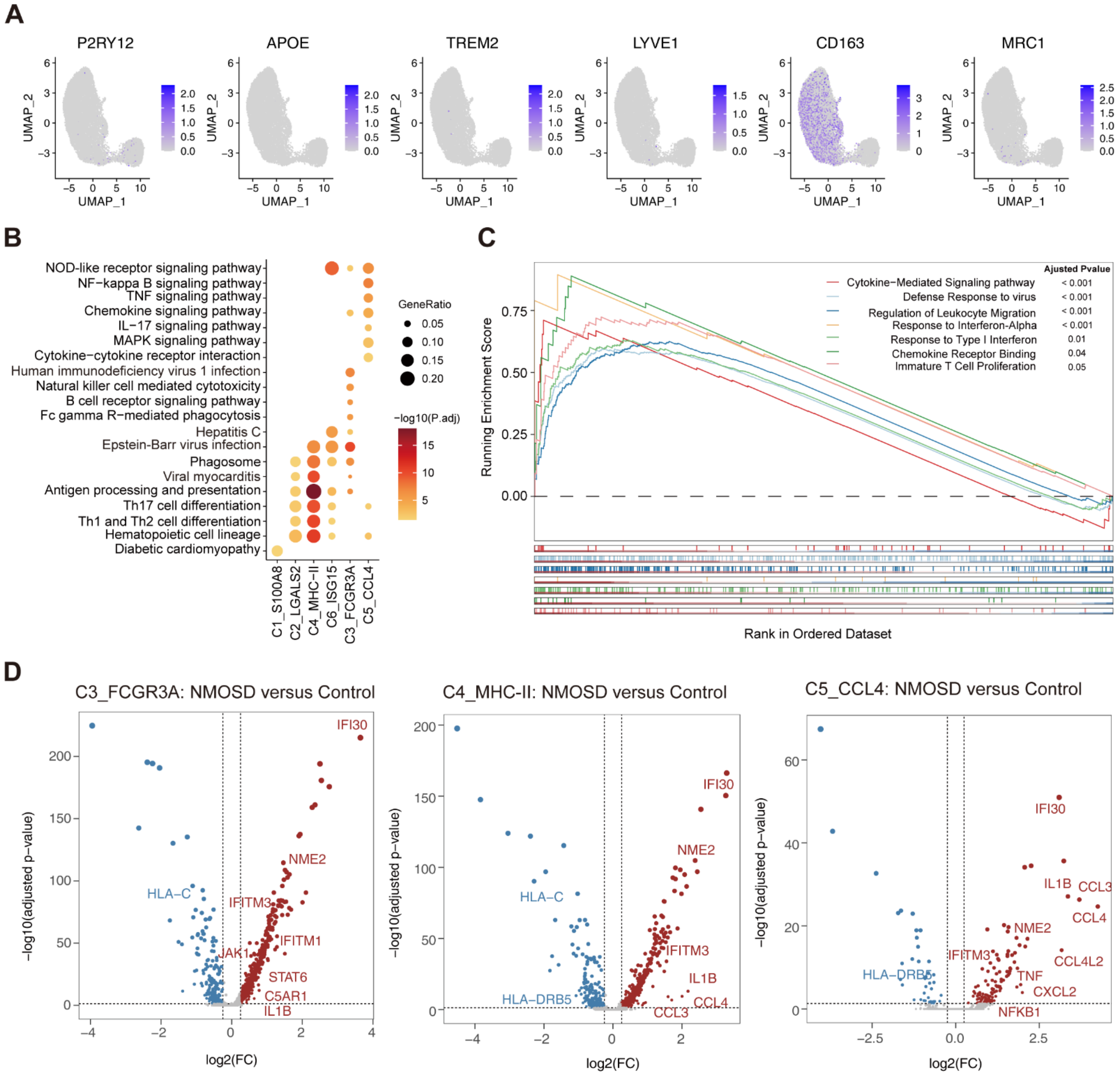

Figure S3. Atypical inflammatory and IFN-I response in the peripheral blood monocytes in NMOSD.

A. The UMAP diagram demonstrated a low expression of marker genes linked to CSF microglia-like and CNS-associated macrophage-like cells within peripheral blood monocytes. B. Dot plot illustrating the KEGG pathways enriched by the highly expressed genes in distinct sub-clusters of blood monocytes. C. GSEA plot illustrating selected significantly enriched KEGG pathways of the peripheral blood monocytes between NMOSD patients and controls. D. Volcano plots depicting representative top DEGs of the blood monocyte sub-clusters.

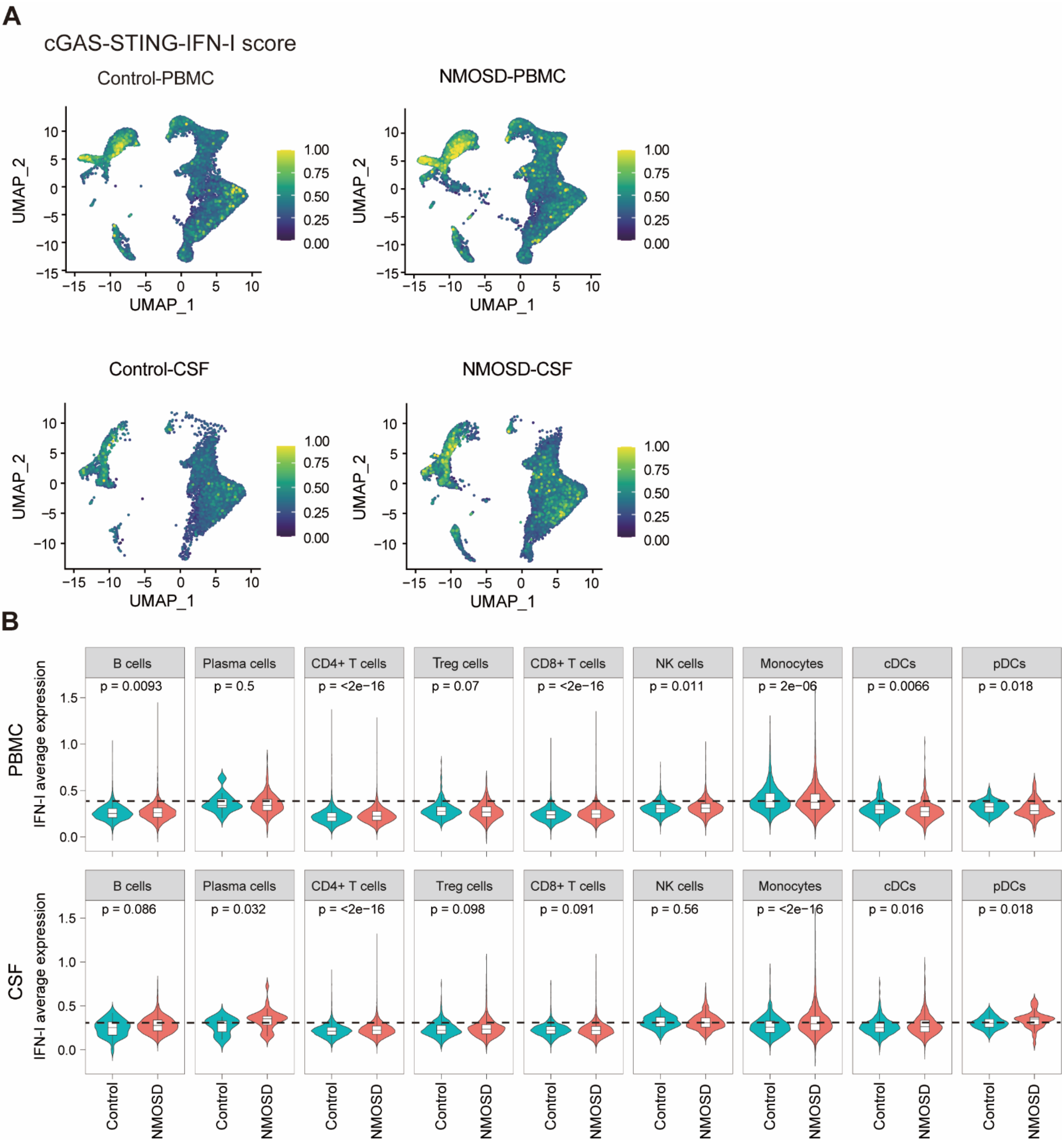

Figure S4. Patients with NMOSD exhibited stronger IFN-I response in myeloid cells.

A. UMAP plot displaying IFN-I gene-set scores of the main cell cluster from different conditions. B. Violin plot visualizing the IFN-I score in the main cell clusters of CSF and blood, comparing NMOSD patients with controls using the Wilcoxon rank-sum test.

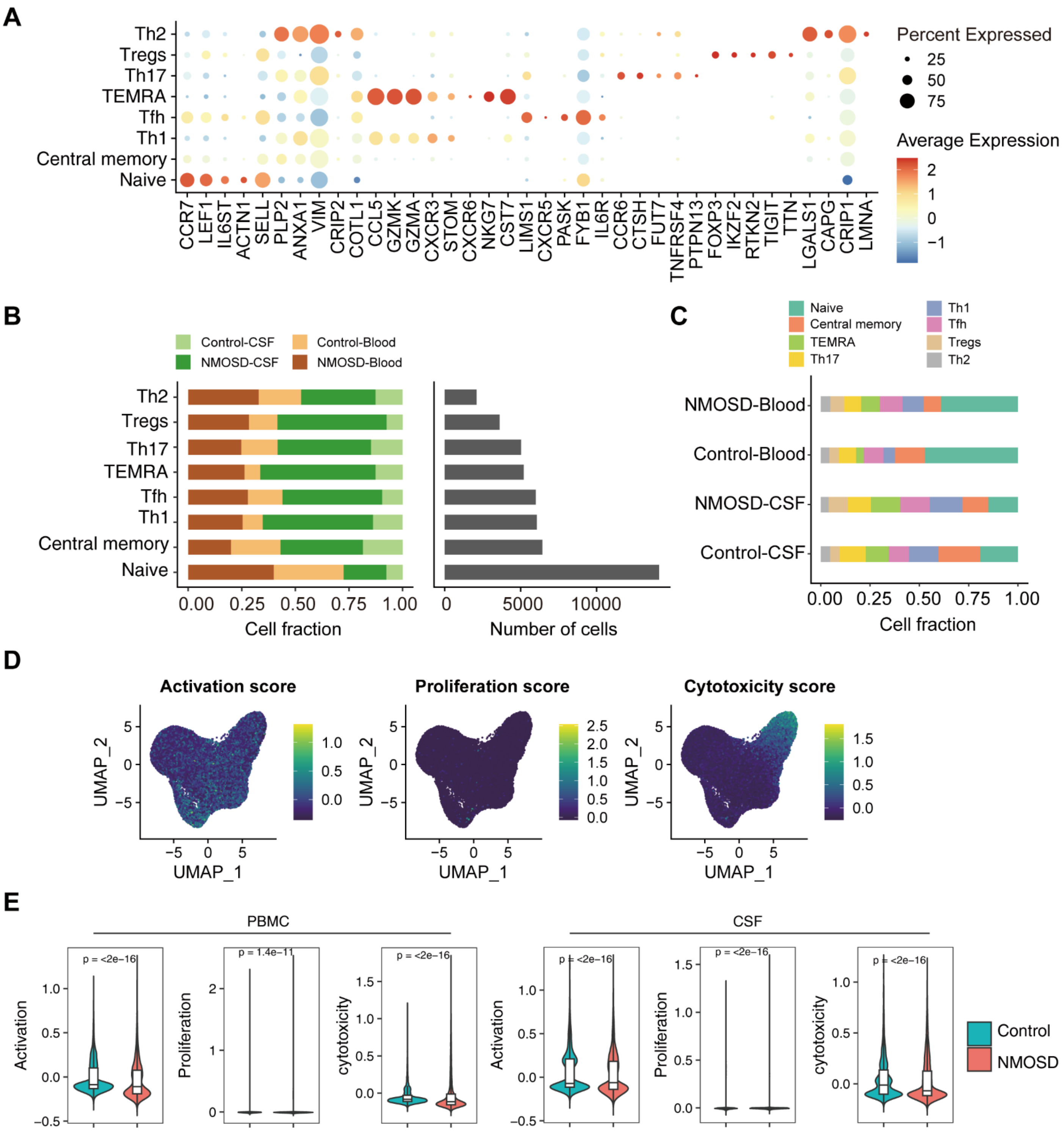

Figure S5. Enhanced autoreactivity of CD4^{+} T cells in patients with NMOSD.

A. Dot plot depicting highly expressed marker genes specific to each sub-cluster. B, C. Proportions of various CD4^{+} T cell sub-clusters identified in peripheral blood and CSF samples from both healthy controls and NMOSD patients. D, E. UMAP and violin plots describing the gene-set scores of activation, proliferation, and cytotoxicity genes in CD4^{+} T cells. These plots highlighted the upregulation of these signatures in CD4^{+} T cells from NMOSD patients compared to controls.

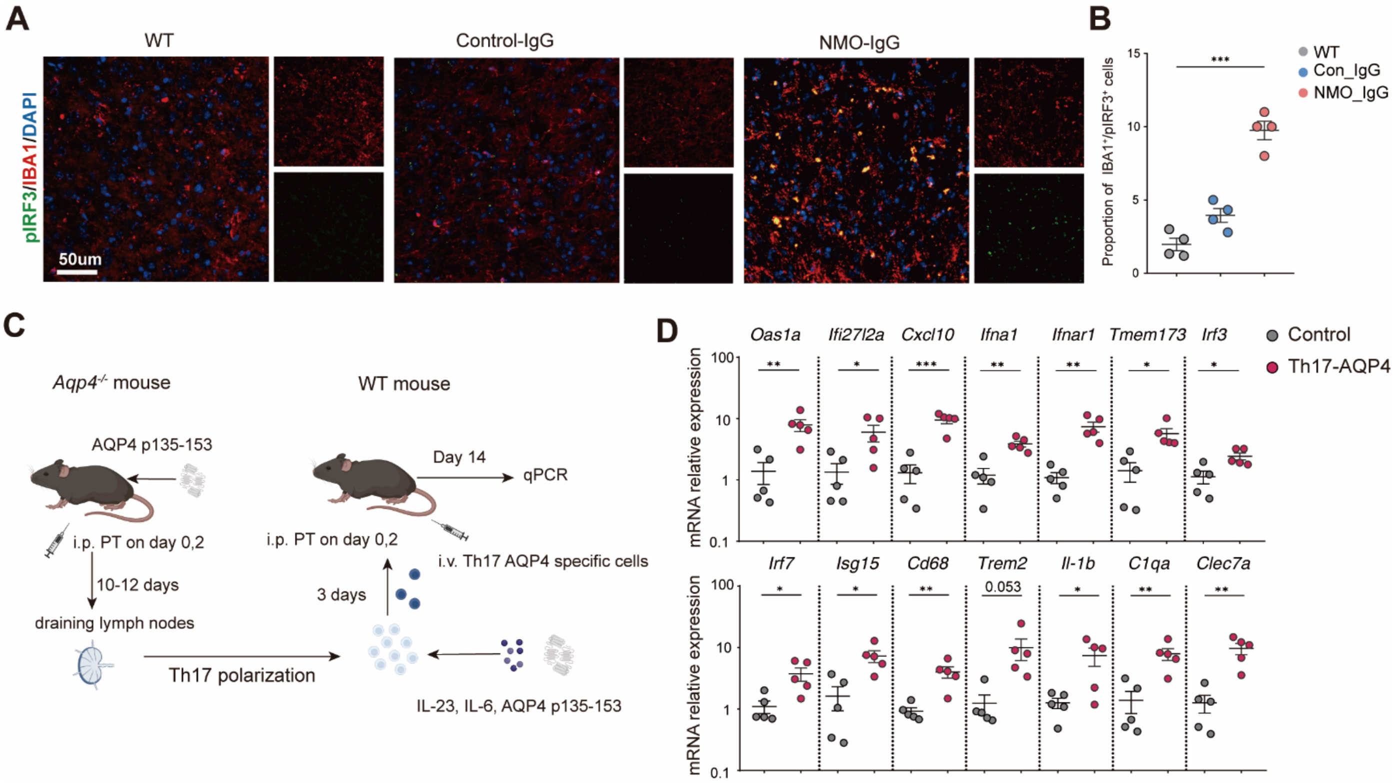

Figure S6. cGAS-STING-IFN-I signaling activation in microglia of NMO-IgG injected models and Th17-AQP4 passive transfer models.

A, B. Representative immunofluorescence staining images and quantification of the proportion of IBA1^{+} microglia with phosphorylated IRF3 (pIRF3) in WT mice, control-IgG treated mice, and NMO-IgG treated mice (n=4/group). C. Schematic diagram illustrating the Th17-AQP4 passive transfer model. D. The expression levels of cGAS-STING- IFN-I related genes in MACS sorted CD11b^{+} microglia of Th17-AQP4 passive transfer model (n=5/group). Data presented as Mean ± s.e.m; *$p < 0.05$, **$p < 0.01$, ***$p < 0.001$, P values were calculated by the Kruskal-Wallis test or Mann-Whitney test.

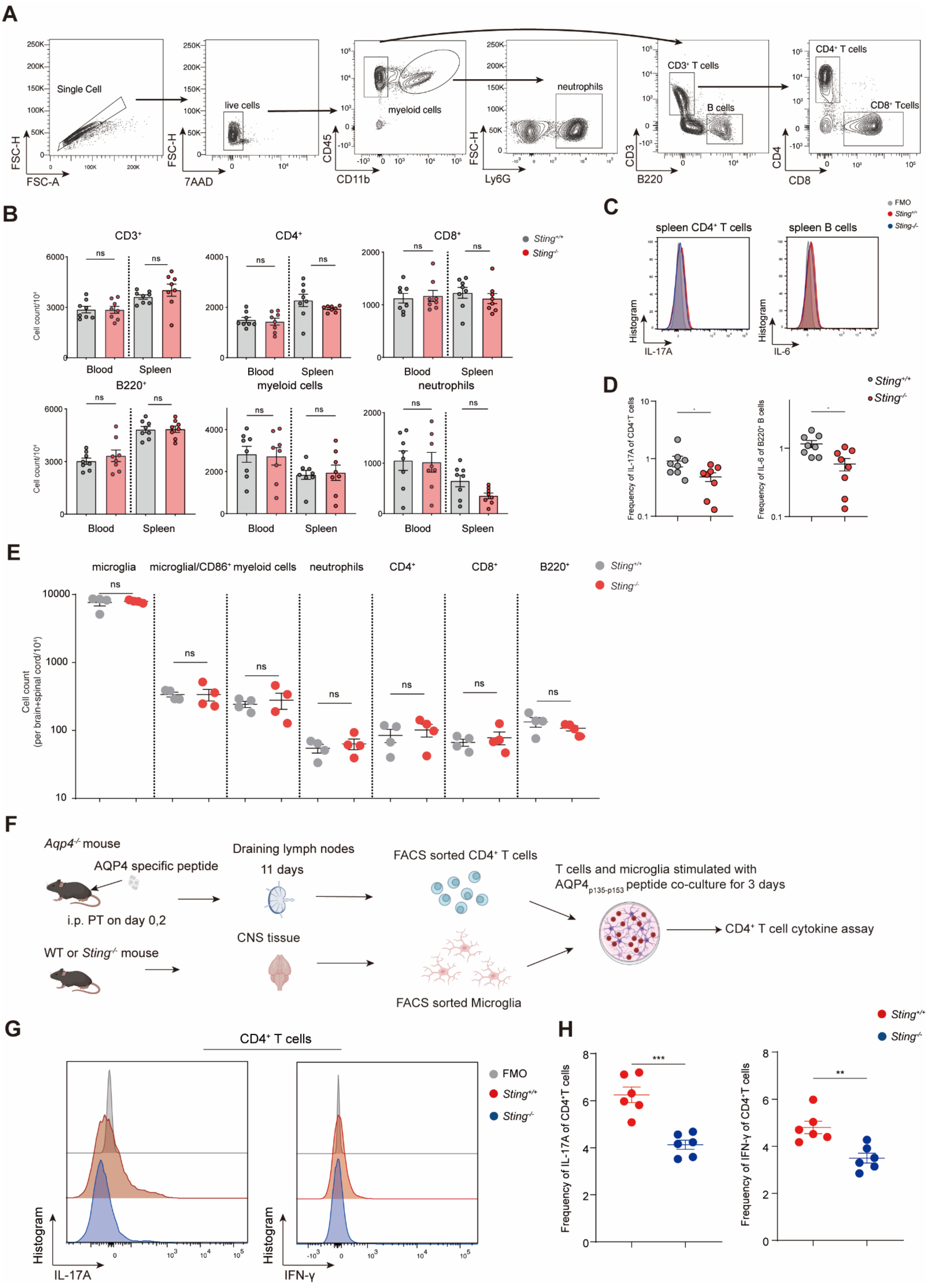

Figure S7. *Sting* deficiency modulated periphery and CNS immune responses in the Th17-AQP4 mice.

A. FACSgating strategy used to identify different cell types in the spleen and peripheral blood. B. Quantitative analysis comparing the T cells and B cells between WT and *Sting*$^{-/-}$ mice in the spleen and peripheral blood (n=8/group). C, D. Intracellular IL-17A in CD4^{+} T cells and IL-6 in B cells were analyzed by FACS between WT and *Sting*$^{-/-}$ mice in the spleen (n=8/group). E. Quantitative analysis of the T cells and B cells within the CNS of WT and *Sting*$^{-/-}$ mice during the course of disease recovery (n=4/group). F. Flow chart depicting the process of in vitro microglia and AQP4-specific T cells. G, H. Representative flow cytometry analysis and statistical evaluation demonstrating the absence of *Sting* in the CNS microglia will reduce the production of IL-17A and IFN-γ by AQP4-specific CD4^{+} T cells in vitro (n=6/group). Statistical comparison between the two groups was conducted using unpaired student's t-test or Mann-Whitney test. The results were presented as Mean ± s.e.m, $^{*}p < 0.05$, $^{**}p < 0.01$, $^{***}p < 0.001$.

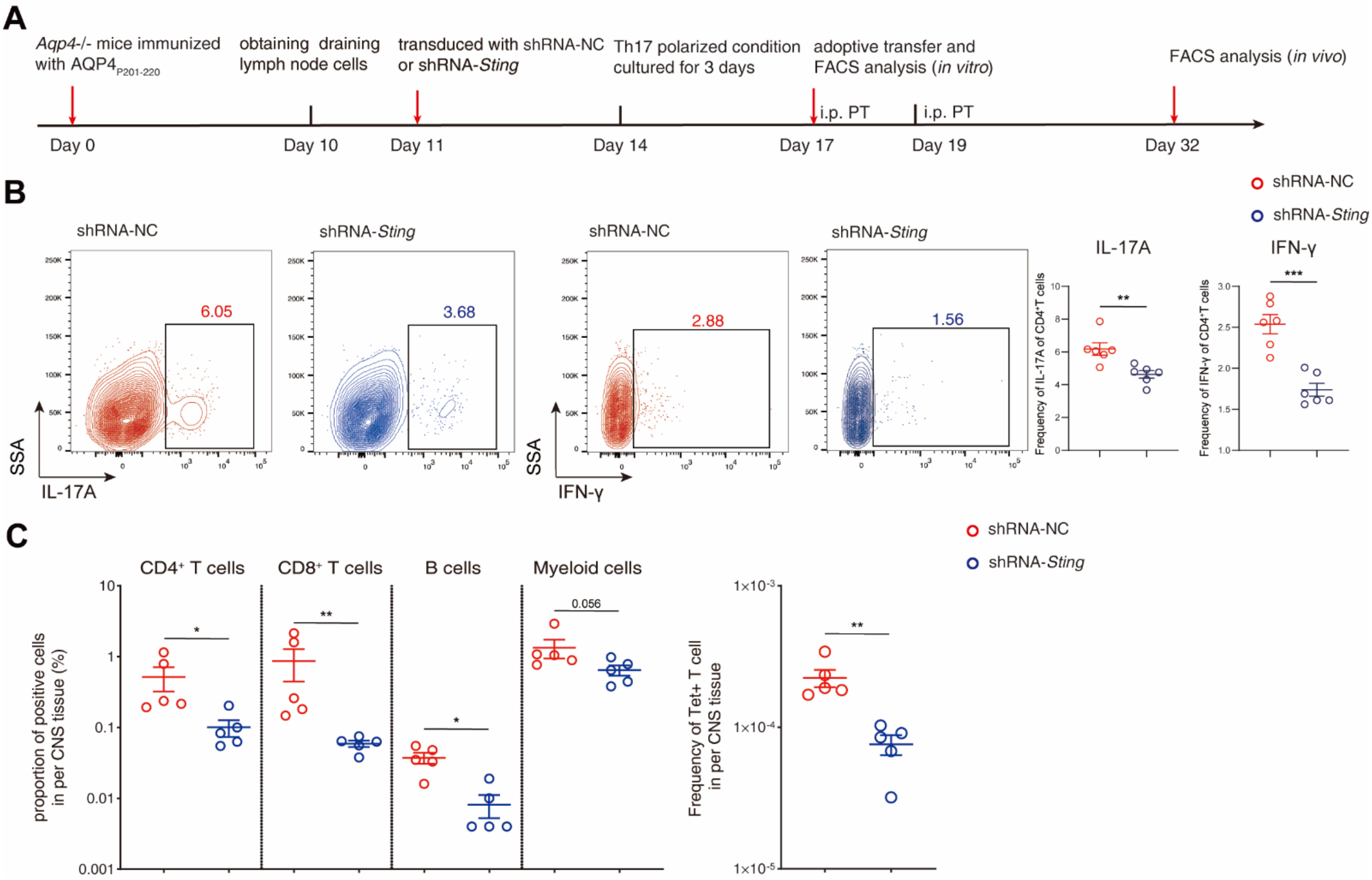

Figure S8. *Sting* knockdown inhibited the inflammatory response of CD4$^+$ T cells and infiltration into the CNS.

A. The flowchart showing the experimental procedure of *Sting* gene knockdown in AQP4 specific immune cells mediated by retroviral transduction. B. *In vitro* cell culture experiments confirmed that knocking down the *Sting* in AQP4-specific T cells effectively inhibit the inflammatory response of CD4$^+$ T cells (n=6). C. Knocking down the *Sting* in AQP4-specific immune cells reduced the infiltration of these cells into the CNS, and the proportion of AQP4 antigen specific T cells also decreased (n=5). Statistical comparison between the two groups was conducted using unpaired student's t-test or Mann-Whitney test. The results are presented as Mean ± s.e.m, *p < 0.05, **p < 0.01, ***p < 0.001.

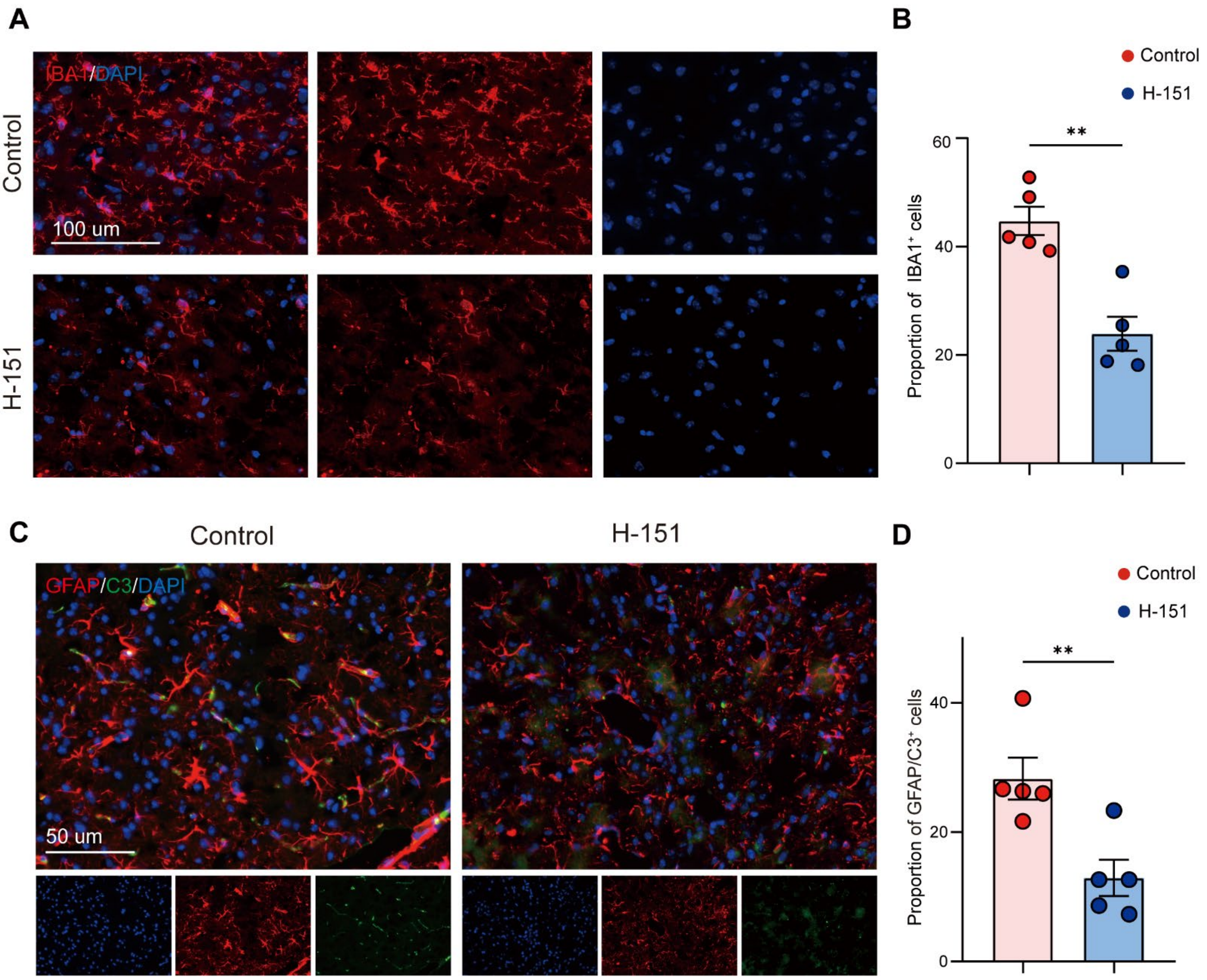

Figure S9. Administration of H-151 treatment effectively attenuated glial activation in NMO-IgG mouse model.

A. Representative immunofluorescence plots depicting alterations in IBA1$^+$ microglia within brain lesions of WT mice and H-151-treated mice at the peak of disease in NMO-IgG mouse model. B. Quantitative analysis of the number of IBA1$^+$ microglia (n=5/per group). C. Representative immunofluorescence map illustrating GFAP$^+$/C3$^+$ astrocytes alteration in the brain lesions of WT mice and H-151-treated mice at the peak of disease in NMO-IgG mouse model. D. Quantitative analysis showing differences in the number of GFAP$^+$/C3$^+$ astrocytes (n=5/per group). Statistical comparison between the two groups was conducted using Mann-Whitney test. The results are presented as Mean ± s.e.m, ** $p < 0.01$.

Table S1. Demographic features of patients with NMOSD and controls enrolled for single cell RNA analysis.

Source	Sample-ID	Age	Sex	Condition	EDSS score	CSF WBC	AQP4-ab
GSE133028	control-1	33	M	Control	N/A	0	N/A
	control-2	27	F	Control	N/A	0	N/A
	control-3	41	M	Control	N/A	1	N/A
GSE138266	control-4	43	F	Control	N/A	1	N/A
	control-5	43	M	Control	N/A	4	N/A
	control -6	32	F	Control	N/A	2	N/A
	control-7	25	F	Control	N/A	1	N/A
	control-8	33	M	Control	N/A	1	N/A
Newly enrolled	NMOSD-1	48	F	NMOSD	4	4	+
	NMOSD-2	58	F	NMOSD	3.5	2	+
	NMOSD-3	35	F	NMOSD	5	2	+
	NMOSD-4	58	F	NMOSD	4	2	+
	NMOSD-5	47	F	NMOSD	6	8	+

Table S2. Demographic characteristics of NMOSD patients and healthy controls enrolled for CSF and plasma cell free dsDNA and plasma 2'3'-cGAMP detection.

	NMOSD (n = 36)	Controls (n = 21)
**Age, mean (SD)***	52.3 ± 16.2	55.1 ± 14.7
**Female, n (%)**	29 (80.6)	18 (85.7)
**Mean years of disease duration (SD)**	6.1±4.4	-
**AQP4-ab positive, n (%)**	31 (86.1)	-
**EDSS at attack (SD)**	4.1±1.4	-
**First onset**	10 (33.3)	-
**Preventive medications prior to this relapse**		
Prednisone	19 (52.8)	-
Rituximab	2 (13.8)	-
Azathiophrine	5 (8.3)	-

Table S3. RT-PCR primer sequence for IFN-related and microglia activation genes.

gene	Forward primer	Reverse Primer
Oas1a	ATTGCCTCCACAGTACGC	GATGACCAGTTCCAAGACG
Ifi27l2a	CCTCCATAGCAGCCAAGA	TGCAACTCCACCTCCATT
Cxcl10	AGCCGTGGTCACATCAG	ATCCCAGCCACTTGAGC
Ifna1	CAGGTGAGGTTGCAGGA	CAGCACATTGGCAGAGG
Ifnar1	CGTAGCCCCTCAGTGTGT	GCCAGCTCCTCCAGTTAGT
Tmem173	GCCTTCGTCTTGCTGGT	TGCAGAGTGTGACTTGGTG
Irf3	CTCCAACAGCCAGCCTATC	CATGTCCTCCACCAAGTCC
Irf7	CCCATCTTCGACTTCAGCA	TGCCCAAAACCCAGGTAG
Isg15	AAGCAGCCAGAAGCAGAC	TTAGGTCCCAGGCCATT
Cd68	CCCTCTTGCTGCCTCTC	GCTCCTTGGTGGCTTACA
Trem2	CCTGACTGGCTTGGTCATC	GCAGGAGAAACTGGTGGAG
Il-1b	AGTTGACGGACCCCAAA	TCTTGTTGATGTGCTGCTG
C1qa	GGAGGGCACTGAGGACTG	CACAGACACAGACGGGGAT
Clec7a	GCCCTGTGAAGCAATGA	GCCTCCAAGGTGAAGATG
β-Actin	CCTCACTGTCCACCTTCC	GGGTGTAAAACGCAGCTC

Table S4. Gene set for cGAS-STING-IFN-I and T cell status score.

cGAS-STING-IFN-I		Th1	Th2	Th17	Activation	Proliferation	Cytotoxicity
ABCA1	LAP3	CCL3	CCR4	CCL20	HLA-DRA	BUB1	ADGRG1
CARD16	LGALS9	CCL4	CCR8	CCR6	ICOS	CCNB1	CX3CR1
CCL8	LY6E	CCL5	CCR10	CTSH	IL2RA	MKI67	EOMES
CCR1	MX1	CXCR3	GATA3	IL17A	ITGA1	PLK1	FCGR3A
CEACAM1	MX2	IRF7	HPGDS	IL1B	ITGAE	TOP2A	FGFBP2
CMPK2	NCOA7	LTA	*MAOA*	IL23A	TNF		GNLY
DDX58	OAS1	STAT1	PPARG	IL23R	TNFRSF4		GZMA
DDX60	OAS2	STAT3	PTGDR2	IL6R			GZMB
DHRS9	OAS3	TBX21	PTGS2	IL6ST			GZMK
EIF2AK2	PARP14	XCL1		IL7R			HOPX
GADD45B	PARP9			KLRB1			IFIT3
GALM	RBCK1			PTPN13			KLRD1
GBP2	RHBDF2			RORA			KLRF1
GBP1	RNF213			RORC			KLRG1
GBP4	RSAD2						NKG7
GBP5	SERPING1						ZEB2
HERC5	SOCS1						ZNF683
IFI16	SP100						
IFI27	SP110						
IFI35	SP140						
IFI44	SPATS2L						
IFI44L	STAT1						
IFIT1	STAT2						
IFIT2	TAP1						
IFIT3	TBK1						
IFITM1	TLR4						
IFITM3	TNFSF10						
IL1RN	TNFSF13B						
IRF4	TRIM22						
IRF7	TRIM69						
IRF8	TYMP						
IRF9	UBE2L6						
ISG15	XAF1						
ISG20	ZC3HAV1						
